# Insight into the metabolic potential and ecological function of a novel Magnetotactic *Nitrospirota* in coral reef habitat

**DOI:** 10.3389/fmicb.2023.1182330

**Published:** 2023-05-17

**Authors:** Yicong Zhao, Wenyan Zhang, Hongmiao Pan, Jianwei Chen, Kaixuan Cui, Long-Fei Wu, Wei Lin, Tian Xiao, Wuchang Zhang, Jia Liu

**Affiliations:** ^1^CAS Key Laboratory of Marine Ecology and Environmental Sciences, Institute of Oceanology, Chinese Academy of Sciences, Qingdao, China; ^2^Laboratory for Marine Ecology and Environmental Science, Pilot National Laboratory for Marine Science and Technology (Qingdao), Qingdao, China; ^3^University of Chinese Academy of Sciences, Beijing, China; ^4^Center for Ocean Mega-Science, Chinese Academy of Sciences, Qingdao, China; ^5^France-China Joint Laboratory for Evolution and Development of Magnetotactic Multicellular Organisms, Chinese Academy of Sciences, Beijing, China; ^6^BGI-Qingdao, BGI-Shenzhen, Qingdao, China; ^7^Aix Marseille University, CNRS, LCB, IM2B, IMM, Marseille, France; ^8^Key Laboratory of Earth and Planetary Physics, Institute of Geology and Geophysics, Chinese Academy of Sciences, Beijing, China

**Keywords:** Magnetotactic bacteria, *Nitrospirota*, metabolic potential, coral ecosystems, ecological functions

## Abstract

Magnetotactic bacteria (MTB) within the *Nitrospirota* phylum play important roles in biogeochemical cycles due to their outstanding ability to biomineralize large amounts of magnetite magnetosomes and intracellular sulfur globules. For several decades, *Nitrospirota* MTB were believed to only live in freshwater or low-salinity environments. While this group have recently been found in marine sediments, their physiological features and ecological roles have remained unclear. In this study, we combine electron microscopy with genomics to characterize a novel population of *Nitrospirota* MTB in a coral reef area of the South China Sea. Both phylogenetic and genomic analyses revealed it as representative of a novel genus, named as *Candidatus* Magnetocorallium paracelense XS-1. The cells of XS-1 are small and vibrioid-shaped, and have bundled chains of bullet-shaped magnetite magnetosomes, sulfur globules, and cytoplasmic vacuole-like structures. Genomic analysis revealed that XS-1 has the potential to respire sulfate and nitrate, and utilize the Wood–Ljungdahl pathway for carbon fixation. XS-1 has versatile metabolic traits that make it different from freshwater *Nitrospirota* MTB, including Pta-ackA pathway, anaerobic sulfite reduction, and thiosulfate disproportionation. XS-1 also encodes both the *cbb*_3_-type and the *aa*_3_-type cytochrome c oxidases, which may function as respiratory energy-transducing enzymes under high oxygen conditions and anaerobic or microaerophilic conditions, respectively. XS-1 has multiple copies of circadian related genes in response to variability in coral reef habitat. Our results implied that XS-1 has a remarkable plasticity to adapt the environment and can play a beneficial role in coral reef ecosystems.

## 1. Introduction

Magnetotactic bacteria (MTB) represent a phylogenetically diverse group of Gram-negative bacteria generally thriving at the oxic-anoxic interface (OAI) of sediments or chemically stratified water columns (Lefèvre and Bazylinski, 2013). The MTB synthesize nanosized magnetite (Fe_3_O_4_) and/or greigite (Fe_3_S_4_) magnetosomes, and use them to orientate in geomagnetic fields (Bazylinski and Frankel, 2004; Bazylinski et al., 2013). Some of them also produced intracellular inclusions in addition to magnetosomes, including lipid storage granules, sulfur globules, polyphosphate (polyP) inclusions, calcium carbonate granules, organic-deficient vacuole structures, and amorphous silica globules, suggesting their metabolic versatility in natural environments and contribution to the biogeochemical cycling of C, N, S, P, and Si (Keim et al., 2005; Lefèvre et al., 2012; Lin et al., 2012; Abreu et al., 2013; Liu et al., 2017; Li et al., 2021). The phylum *Nitrospirota* is the most exceptional but rarely identified lineage of MTB. They are known to produce hundreds of bullet-shaped magnetosomes that are synthesized within single giant-rod-shaped cells (Vali et al., 1987; Spring et al., 1993). The phylum occupies an independent phylogenetic position on the tree of MTB.

To date, *Nitrospirota* MTB of high morphological and phylogenetic diversity have been widely reported in freshwater environments, and to a smaller extent in high temperature and acidic habitats (Spring et al., 1993; Flies et al., 2005; Lefèvre et al., 2010; Lin et al., 2011, 2012, 2020; Uzun et al., 2020; Zhang et al., 2021). Because of the current inability to cultivate *Nitrospirota* MTB in laboratory settings, most of the physiological information on the phylum is speculation based on microscopic observations of cell ultrastructure combined with genetic information. *Ca*. Magnetoovum mohavensis LO-1 is ovoid in shape (3.50 × 2.70 μm), and has hundreds of magnetosomes arranged in several bundles (Lefèvre et al., 2011a). In addition to magnetosomes, its cells generally contain two other types of inclusions including sulfur globules and carboxysomes containing the potential CO_2_-fixing enzyme RubisCO, suggesting an autotrophic lifestyle for LO-1 (Lefèvre et al., 2011a). This conclusion was strengthened by the fact that the closely related strain *Ca.* Magnetoovum chiemensis contains all the genes involved in the Wood–Ljungdahl pathway (WL pathway) for carbon fixation (Kolinko et al., 2016). In addition, the two giant-rod morphotypes *Ca.* Magnetobacterium bavaricum and *Ca.* Magnetobacterium casensis encode proteins for oxidizing sulfide to elemental sulfur, which is consistent with their high intracellular sulfur content. Advanced electron microscopy and metagenomic analysis have recently shown that *Nitrospirota* MTB strain WYHC-5 contains the *silicatein-1* gene in its genome, and accumulates silicon particles intracellularly (Li et al., 2022). Among all the identified *Nitrospirota* MTB, three vibrioid-shaped species have been reported including MHB-1 (1.00 × 2.00 μm) from a human-affected lake area (Bremen, Germany; Flies et al., 2005), HSMV-1 (0.30 × 1.40 μm) from hot spring sediments (Nevada, the USA; Lefèvre et al., 2011b), and LBB01 (0.50 × 2.00 μm) from a freshwater lake (Shatura district, Russia; Uzun et al., 2022). Although significantly smaller in cell size compared with other members, they still contained numerous magnetosomes (12–60) within each cell.

In contrast to the extensive studies that demonstrate broad biogeographic distribution and high metabolic plasticity among *Nitrospirota* MTB in terrestrial ecosystems, *Nitrospirota* MTB living in marine ecosystems was discovered only recently (Qian et al., 2019a). These shared <94% gene identity with any available 16S rRNA gene sequences in the NCBI database, and thus represent a novel genus-level lineage of *Nitrospirota* MTB. Cells of the marine species are large and ovoid in shape (4.00 × 5.20 μm), and contain thousands of bullet-shaped magnetosomes arranged in bundles of multiple chains. In addition, the cells are filled with sulfur globules that are considered to be energy storage granules (Qian et al., 2019a). Although the potential for iron- and sulfur-based metabolism was evident using transmission electron microscopy (TEM) analysis, the role of marine *Nitrospirota* MTB in biogeochemical cycling remains unclear because of the lack of genomic data.

Tropical coral reefs are the marine equivalent of terrestrial tropical rainforests in terms of their ecologic importance (Reaka-Kudla, 1997). Increasing attention has been directed to the bacterial populations in coral-associated sediments. A recent gene- and genome-centric metagenomics study revealed considerable phylogenetic and functional diversity in the microbial community of coral reef sediments of the Xisha Islands (Dong et al., 2023), and pointed to an essential role of sediment bacteria in biogeochemical recycling in coral reef ecosystems, via carbon fixation and the autotrophic respiration of sulfur compounds. It is interesting to note that, we recently described the morphological and phylogenetic diversity of MTB in coral reef sediments of the Xisha Islands and in the Drummond Island intertidal zone (coral reef area) (Chen et al., 2016; Teng et al., 2018b). Members of a peculiar morphotype, multicellular magnetotactic prokaryotes (MMPs), were found to be present at nine stations encompassing most of the coral reef area of the Xisha Islands (Teng et al., 2018b), suggesting the potentially broad roles of these magnetotactic microorganisms for coral reef ecosystems. The diversity of coral-associated MTB and their contribution to biogeochemical cycling is worthy of attention, yet remains to be fully explored.

This study is the first to report the genome of a marine *Nitrospirota* MTB obtained from a coral reef area, and provides a detailed description of their phylogeny, cell morphology, and genome-based metabolic potential. We also undertook a metabolic comparison between marine and freshwater *Nitrospirota* MTB based on genomic information. We have addressed here the adaption strategies and ecological roles of magnetotactic microorganisms in coral reef habitats.

## 2. Materials and methods

### 2.1. Sampling and bulk collection of MTB

Sediment samples were collected from a coral reef area in the Xisha Islands (112°20′E, 16°57′N), in the South China Sea. The top 1 cm of sediment was discarded, and the underlying 2–5 cm was collected into 500-ml plastic bottles. Each bottle contained approximately 200 ml sediment and 150 ml *in situ* seawater. Samples were stored undisturbed in darkness at room temperature (~25°C) for almost 5 years. Magnetotactic bacteria in the sediments were magnetically enriched by attaching the south pole of permanent magnets outside the bottles (Pan et al., 2008), and were purified using the ‘racetrack’ method as described in previous studies (Wolfe et al., 1987).

### 2.2. Optical and electron microscopy

The abundance and motility of cells were analyzed using the hanging drop method using differential interference contrast (DIC) microscopy (Olympus BX51 equipped with a DP80 camera system; Tokyo, Japan; Schüler, 2002). Magnetically enriched samples were used for the abundance calculation. The precise abundance of cells in the sediments was calculated based on the formula: *C*_(cell)_ = (*N*_(count)_ / *V1*_(drop)_) × *V2*_(collection)_/*V0*_(sediments)_. The volume of sediments (*V0*) was calculated by the bottom area of the sample bottle and the depth of sediment in the bottle. The volume of droplet (*V1*) and collected liquid (*V2*) were controlled by pipettes. The cell velocity was measured using the MTrackJ Plugins of ImageJ software (Meijering et al., 2012). To describe the morphological and structural characteristics of cells, magnetosomes, and other inclusions, TEM observations were performed using a Hitachi HT7700 TEM (Hitachi Ltd., Tokyo, Japan) operating at 100 kV. A JEM-2100 TEM (JEOL, Tokyo, Japan) operating at 200 kV and equipped with an X-Max energy-dispersive X-ray spectrometry (EDXS) system (Oxford Instruments, Oxford, UK) was used for high-resolution TEM (HRTEM), selected-area electron diffraction (SAED), and chemical analysis. The average dry weight of iron per XS-1 cell was calculated based on the formula: W = (ρ_1_ × nV × S) / M × 100%. ρ_1_: the density of Fe_3_O_4_ (5.18 g/cm^3^; Vella and Emerson, 2019). n: the average number of magnetosomes in an individual XS-1 cell. V: the average volume of magnetosome (nm^3^), which was roughly disassembled as a combination of a cone and a cylinder. S: the mass fraction of iron in magnetite crystal. M: the average dry weight of XS-1 cells. According to Cooke and Kuntz (1974), the intracellular water content is approximately 80% and the bacteria cell density is 1.10 g/cm^3^.

### 2.3. Genomic DNA extraction and whole-genome amplification

The XS-1 cells were purified and sorted using a TransferMan ONM-2D micromanipulator equipped with CellTram Oil manual hydraulic pressure control system (IM-9B), and an Olympus IX51 microscope (Tokyo, Japan). The methods used have been described in more detail previously (Chen et al., 2015). Cells were transferred into 3 μl PBS and treated with repeated freeze/thaw cycles. Whole-genome amplification (WGA) was performed using the REPLI-g Single Cell kit (Qiagen, Hilden, Germany) according to the manufacturer’s instructions. WAG products were diluted and subsequently used as the template for 16S rRNA gene amplification. The universal bacterial primers 27F (5′-AGA GTT TGA TCC TGG CTC AG-3′) and 1492R (5′-GGT TAC CTT GTT ACG ACT T-3′) were used for the polymerase chain reaction (PCR). The PCR products were purified (MiniBEAST Agarose Gel DNA Extraction Kit; Takara, Japan), cloned into pMD18-T vector (Takara, Shiga, Japan), and transferred into competent *Escherichia coli* (*E. coli*) DH5α cells (Takara, Japan). Clones were randomly selected for full-length sequencing (Beijing RuiBiotech, China). The 16S rDNA sequences obtained were analyzed using the BLAST search program on the NCBI website.^1^

### 2.4. Fluorescence *in situ* hybridization

For fluorescence *in situ* hybridization (FISH) analysis, a species-specific oligonucleotide probe (XS1-822; 5′-AAC CTT ACG AGC CTA CAC CT-3′; nucleotide position 822 to 841) was designed to target the 16S rRNA gene of strain XS-1. The specific probe was designed by the probe design software Primer (Version 5.0; Singh et al., 1998), and its specificity was evaluated using the online probe evaluation tools ProbeMatch (Cole et al., 2009). The universal bacterial probe EUB338 (5′-GCT GCC TCC CGT AGG AGT-3′) was used as a positive control probe. Probe EUB338 was synthesized and labeled fluorescently with fluorescein phosphoramidite FAM at the 5′ end, while probe XS1-822 was synthesized and labeled fluorescently with hydrophilic sulfoindocyanine dye Cy3 at the 5′ end. *E. coli* cells were used as an internal control. We performed gradient dilution of the *E. coli* solution, and mixed it with XS-1 cells, respectively. After stained with crystal violet, we checked the mixture under microscopy and chose the one with approximately 1:1 number of XS-1 and *E. coli* in the field of view. FISH was carried out as described previously (Pan et al., 2008).

### 2.5. Genome assembly and annotation

Paired end 100 bp (PE100) libraries were constructed from the WGA product, using the MGI Easy FS DNA Library Prep Set kit (MGI, Shenzhen, China), according to manufacturer’s instructions. The genome was sequenced using the DNBSEQ-T1 platform (BGI-Qingdao, China). After quality trimming and filtering using SOAPnuke version 2.1.6 (Chen et al., 2018), the clean reads were assembled using Megahit version 1.2.8 (Li et al., 2015), with k-mer sizes from 27 to 255 by step 20. The metaWRAP version 1.2.1 pipeline was used for metagenome binning, refinement, and reassembly using default parameters to select the genome (Uritskiy et al., 2018). The quality of the XS-1 genome was assessed using QUAST version 5.0.2 (Gurevich et al., 2013), and genomic completeness and contamination were estimated using CheckM version 1.1.3 (Parks et al., 2015).

Gene prediction and annotation were performed using Prokka (version 1.14.6; Seemann, 2014). Protein function was annotated based on the Kyoto Encyclopedia of Genes and Genomes (KEGG) database (Kanehisa et al., 2017). KEGG pathway annotation and mapping was performed using BlastKOALA and KEGG mapper (Kanehisa et al., 2016). For comparative genomic analysis, a total of seven representative *Nitrospirota* MTB genomes and three marine MTB genomes were obtained from the NCBI GenBank database. The identification, annotation, and visualization of magnetosome gene clusters (MGCs) were performed using MAGcluster (Ji et al., 2022) with manual inspection.

### 2.6. Phylogenetic analysis

The genome-based phylogeny was determined based on the Genome Taxonomy Database (GTDB) taxonomy (Parks et al., 2018, 2020). *Nitrospirota* MTB genomes having genome completeness >85%, obtained from the NCBI database, were used as reference genomes. The identification of marker genes, and multiple sequence alignment based on the bac120 marker set, was conducted using GTDB-Tk version 1.6.0 (Chaumeil et al., 2019). A phylogenomic tree rooted with *Deferribacter desulfuricans* SSM1 and *Deferribacter autotrophicus* SL50 was inferred using IQ-TREE (Nguyen et al., 2015) with default parameters. The average amino acid identity (AAI) values were calculated using CompareM software.^2^

The reference sequences for 16S rDNA and KaiC (amino acid sequences) were downloaded from the NCBI database. All related sequences were aligned using BioEdit Alignment Editor software (version 7.0.5.3), using the CLUSTAL W multiple method. Alignments were corrected and trimmed manually. The phylogenetic tree based on 16S rDNA were constructed using MEGA version 6.0 and the neighbor-joining method (Tamura et al., 2013), while the KaiC phylogenetic tree was constructed using the maximum likelihood method.

## 3. Results

### 3.1. Identification of a novel *Nitrospirota* MTB

**Figure 1:**
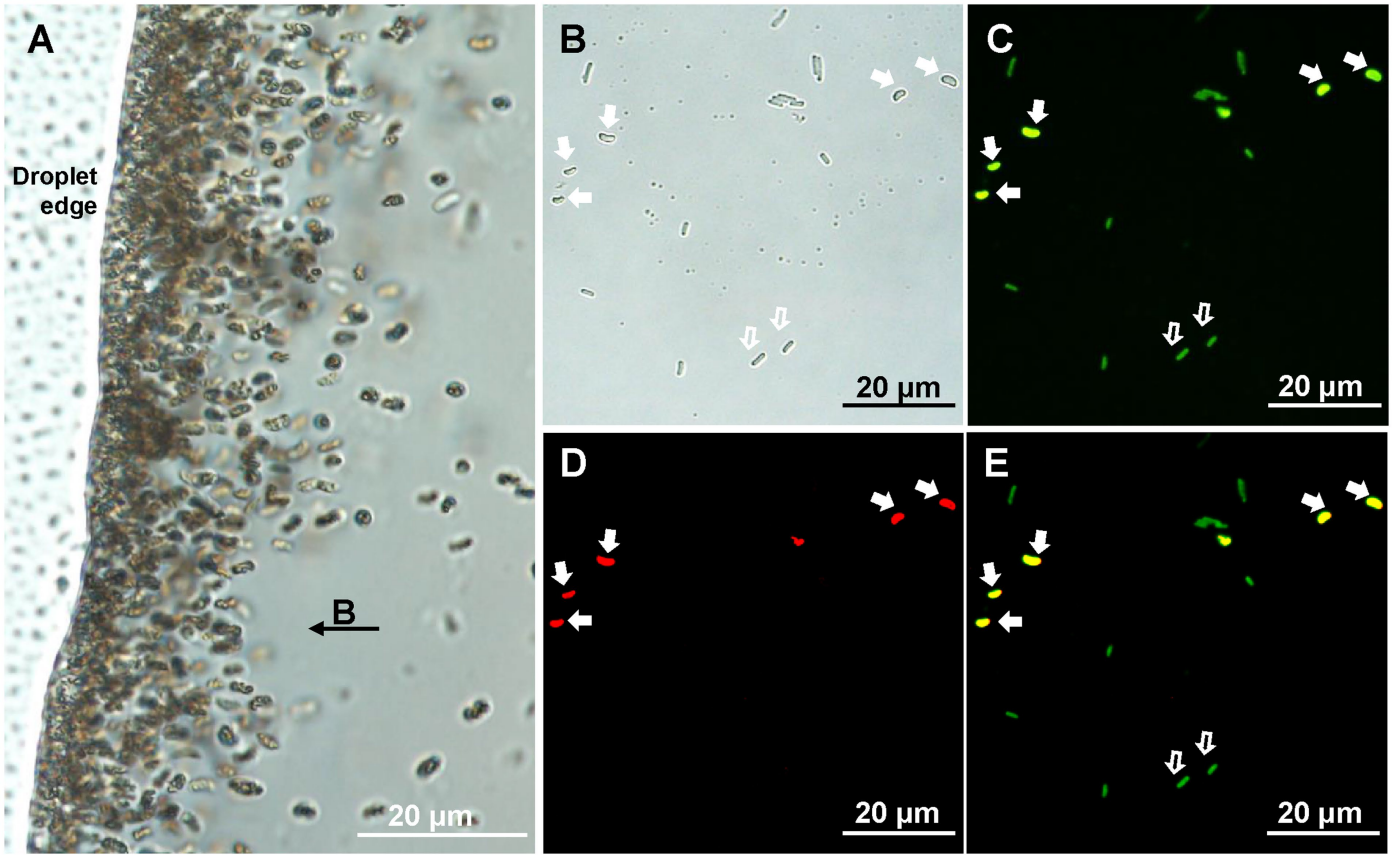
Identification of XS-1. **(A)** Light microscopy image of XS-1 enrichment. Note that the applied field direction is from right to left, and the bacteria were concentrated at the left edge of the water droplet. **(B-E)** Specific detection of XS-1 using fluorescence *in situ* hybridization analysis. Optical microscopy image **(B)** and fluorescence microscopy images show the same field of view where cells were, respectively, stained with bacterial universal probe EUB338 **(C)** and with species-specific probe XS1-882 **(D)**. The superposition image of the two hybridizations is shown in **(E)**. The cells of XS-1 are indicated by solid white arrows and the *E. coli* cells (internal control) are indicated by hollow white arrows.

After 5 years of storage in the laboratory at room temperature, small vibrioid cells were the dominant MTB morphotype (10^3^–10^4^ cells/cm^3^) in the coral reef sediment samples collected from the Xisha Islands (Figure 1A). Cells of the same morphotype were selected using micromanipulation for phylogenetic analyses. Their full-length 16S rRNA gene sequences were amplified, cloned, and sequenced. The 21 tested clones shared 99.00–100.00% sequence identity and formed one operational taxonomic unit (OTU). The closest match in the NCBI-nr database (90.85% identity) was to an uncultivated *Nitrospirota* MTB (GenBank accession no. KY921893). The specific 16S rDNA FISH probe XS1-822 was designed to target this OTU. FISH observations showed that all small vibrioid cells were stained with both the general bacterial probe EUB338-FAM and the XS1-822-Cy3 probe, while the internal control cells of *E. coli* only stained with EUB338-FAM (Figures 1B–E). These cells are referred to as strain XS-1.

### 3.2. Motility, magnetotaxis, and ultrastructure of XS-1

In the presence of an applied magnetic field, freshly collected XS-1 cells displayed polar north-seeking motility in a hanging drop, and had an average velocity of 39.25 ± 5.10 μm/s (*n* = 31; Supplementary Movie S1). The behavior of cells at the edge of the droplet could be divided into three phases. (1) The cells first moved away from the edge of the droplet against the magnetic field direction at an average velocity of 21.77 ± 4.41 μm/s (*n* = 8). (2) Cell motility paused briefly (0–0.45 s). (3) The cells then returned to the droplet edge along the magnetic field direction at a greater velocity (30.22 ± 4.08 μm/s; *n* = 8; Supplementary Movie S2). Similar behavior called “ping-pong” motion were common in MMPs, which was triggered by magnetic field strength (Greenberg et al., 2005), and was postulated to be related with multicellular structures (Qian et al., 2019b). The ‘ping-pong’ motion is occasionally observed in unicellular MTB (Teng et al., 2018a; Zhang and Wu, 2020). The motion of XS-1 was distinguished from “ping-pong” motion by a slow escape motility and a faster return. Since this behavior of the individual cell was accompanied cell aggregation at the droplet edge as showing in Supplementary Movie S1, we termed this phenomenon “volcano motility behavior.”

**Figure 2:**
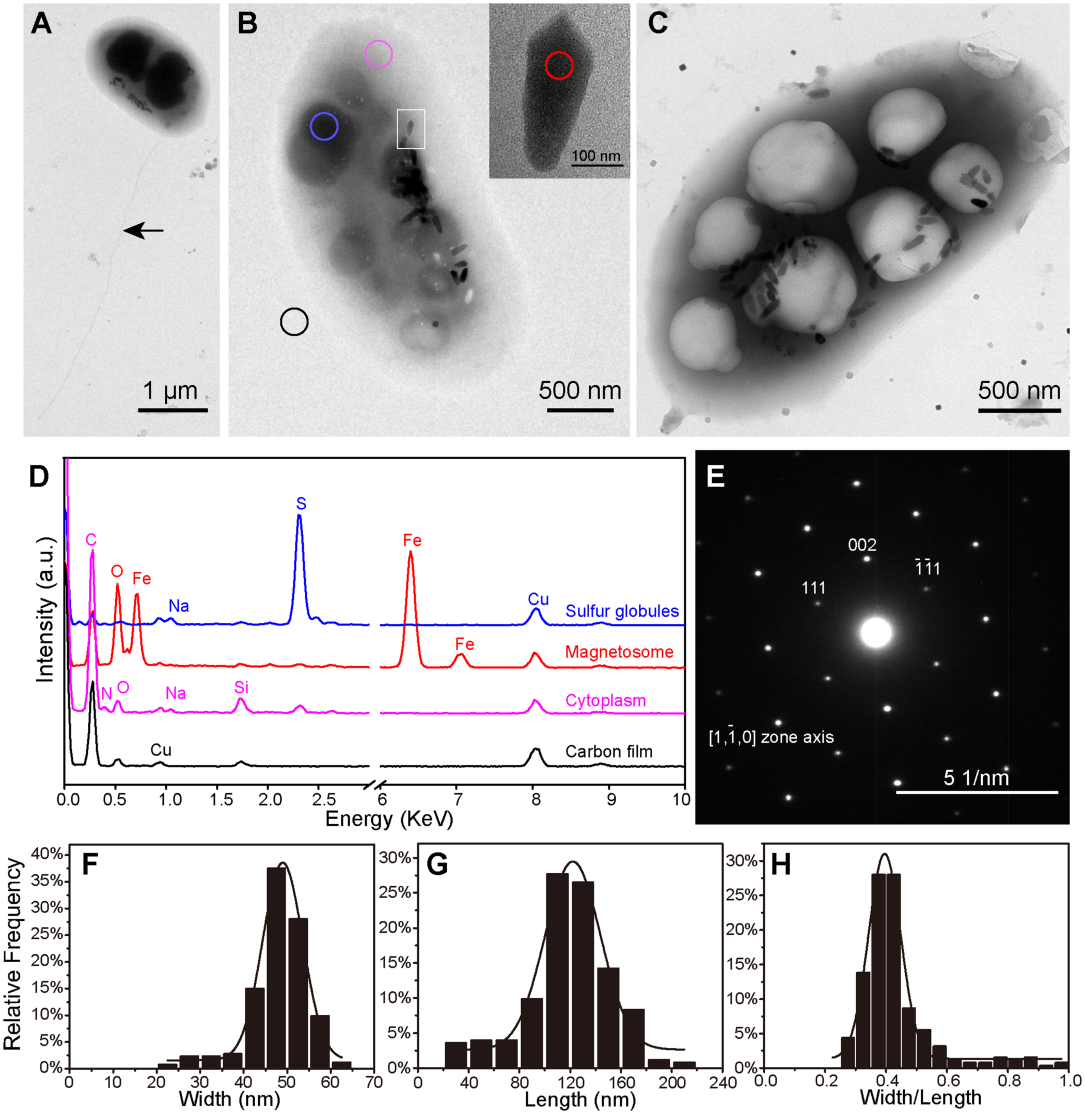
Ultrastructure and intracellular inclusions of XS-1. **(A–C)** Transmission electron micrographs showing the XS-1 cells with a flagellum (**A**, indicated by the black arrow), sulfur globules (**B**, blue circle) and cytoplasmic vacuole-like structures **(C)**. The inset in **(B)** shows a magnified image of the bullet-shaped magnetosome indicated by the white rectangle. **(D)** EDXS analysis of various regions of the cell in **(B)**: sulfur globules (blue), magnetosome (red), cytoplasm (pink), and Carbon film (black). a.u., arbitrary unit. **(E)** Electron diffraction image of the magnetosome. **(F–H)** Frequency histograms of the width **(F)**, length **(G)**, and the shape factor (width/length ratio; **H**) for bullet-shaped magnetosomes in XS-1 cells.

TEM analysis showed that the vibrioid cells were 2.50 ± 0.44 × 1.29 ± 0.19 μm (*n* = 32) in size. A single polar flagellum, approximately four-fold longer than the cell length, was observed (Figure 2A). Each cell contained 26 ± 8 (*n* = 64) magnetosomes (Figures 2B,C). Most were bullet-shaped, 110.00 ± 40.17 nm long × 46.19 ± 7.66 nm wide at the widest point, and had a shape factor (width/length) of 0.43 ± 0.12 (*n* = 253; Figures 2F–H). The magnetosomes were in most cases loosely organized in one bundle arranged approximately along the long axis of the cell. Intriguingly, immature irregular-shaped crystals accounting for 19.13% ± 7.44% of all magnetosomes were scattered in various regions of the magnetosome bundle (Supplementary Figure S1). EDXS and SAED analyses indicated that both the bullet-shaped and the immature crystals were composed of magnetite (Fe_3_O_4_; Figures 2D,E). According to the volume of magnetite crystals, statistical analysis indicates that the average dry weight of iron per XS-1 cell is 2.30% of the cell biomass. TEM observations also revealed the considerable presence of electron dense inclusions and/or electron transparent vacuole-like structures in XS-1 cells (Figures 2B,C). The electron dense inclusions were sulfur-rich (Figure 2D), while the electron transparent vacuole-like structures had the same EDXS spectrum signals with cytoplasm. Among the observed cells, 62.29% (*n* = 122) contained only sulfur globules, 22.95% contained only vacuole-like structures, and 7.38% contained both inclusion types. In most cases, these inclusions occupied a large proportion (32.07% ± 6.31%) of the cell volume, suggesting they are physiologically important.

### 3.3. High-quality genome assembly

Metagenomic sequencing of the micromanipulation-targeted WGA products resulted in 262.93 M reads; these were quality trimmed and assembled into contigs. *De novo* assembly and binning of the contigs enabled reconstruction of a genome of 6.01 Mb in size (Supplementary Table S1), which is much larger than other *Nitrospirota* MTB genomes (1.91–4.23 Mb; Lin et al., 2011; Kolinko et al., 2016; Lin et al., 2020; Zhang et al., 2021; Li et al., 2022), except for the 6.30 Mb genome of *Ca.* Magnetobacterium bavaricum (Kolinko et al., 2016). The draft genome of XS-1 was 97.27% complete with 3.64% contamination, and contained 4,858 protein coding sequences (CDSs), 47 tRNA and one copy each of 5S, 16S, and 23S rRNA. The 16S rRNA gene sequence was 99.30–99.90% identical to the amplified ones described above. Consequently, the genome could be classified as a high-quality draft MAG, following the reporting standards of Bowers et al. (2017), and represent the dominant morphotype of MTB in this coral reef sedimental sample.

### 3.4. Phylogenetic analysis

**Figure 3:**
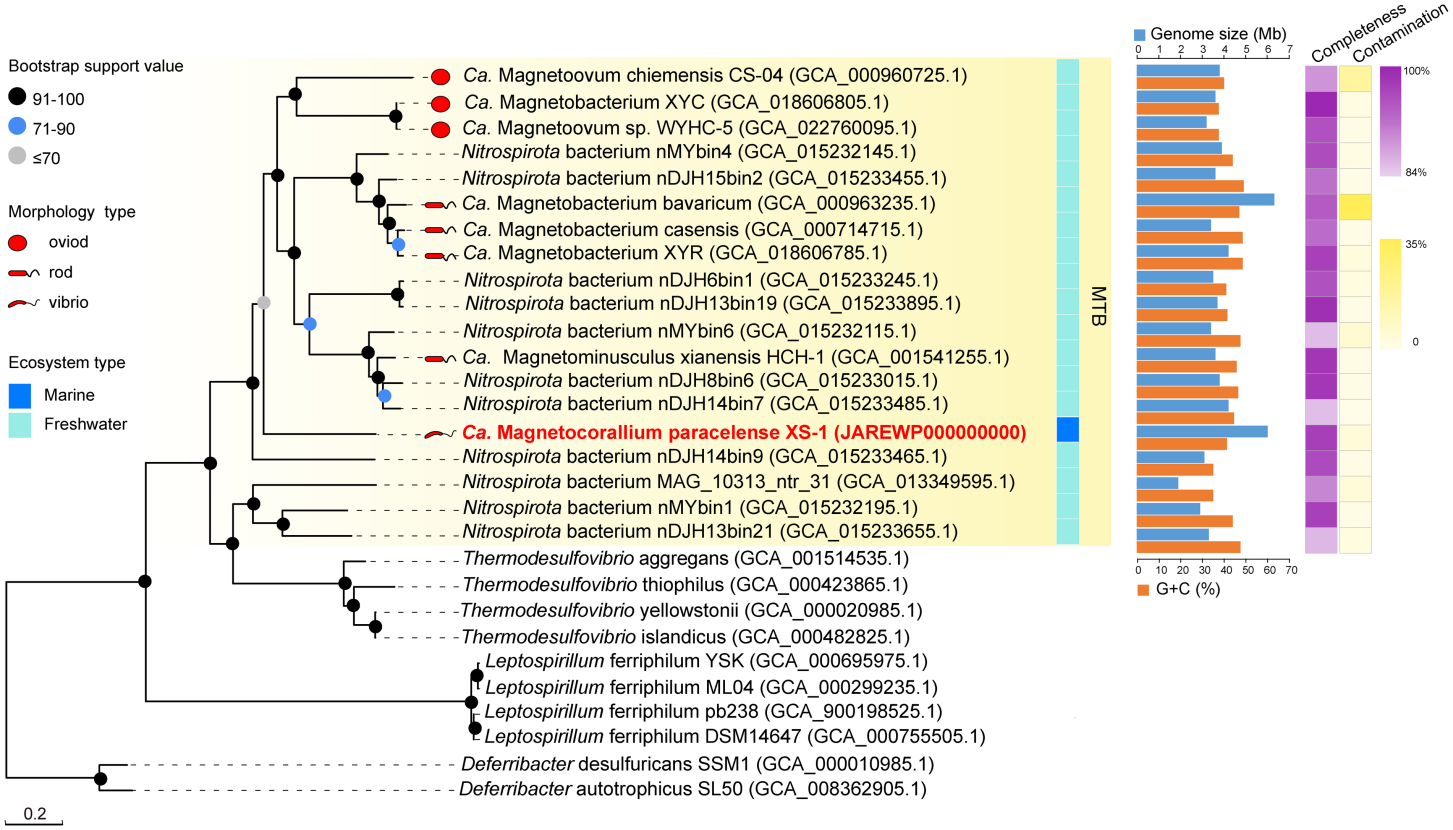
Maximum likelihood phylogenetic tree of representatives of MTB of the phylum *Nitrospirota*. The multiple sequence alignment consisting of 120 concatenated phylogenetic marker genes contained 18 metagenome-assemble genomes (MAGs) of MTB from the Genome Taxonomy Database (GTDB), the MAG of *Ca*. Magnetocorallium paracelense XS-1, four genomes of *Thermodesulfovibrio* spp. and the other genomes of *Nitrospirota*. All MAGs of *Nitrospirota* MTB were estimated to be >85% complete. Bootstrap values at nodes are given as percentages of 1,000 replicates.

For phylogenetic analysis, a concatenated alignment of the 120 bacterial single copy proteins was used for maximum likelihood tree reconstruction using GTDB-Tk (Chaumeil et al., 2019). All genomes of identified *Nitrospirota* MTB formed a clade, a sister taxon to *Thermodesulfovibrio* spp., with 100% bootstrap support (Figure 3). In contrast to other *Nitrospirota* MTB, which clustered together within a well-supported subclade (93% bootstrap), XS-1 formed a distinct subclade. Based on the genomic sequences, XS-1 shared 55.75–57.26% amino acid identity (AAI) with other *Nitrospirota* MTB (Supplementary Table S2), which is well below the cut-off level (65% AAI) for genus delineation (Konstantinidis et al., 2017; Jain et al., 2018). Although phylogenetic analysis of 16S rRNA gene sequences in Supplementary Figure S2 appeared to provide evidence that XS-1 is closely related to the uncultured *Magnetoovum* group, including the oceanic members (GenBank accession nos. KY921892.1, KY921893.1, MK073023.1, and MK203828.1), the association was not strongly supported by bootstrap analysis (46%). Indeed, all *Nitrospirota* sequences used for the phylogenetic tree reconstruction were < 91% similar to the 16S rRNA gene sequence of XS-1, so failing to meet the proposed genus-level threshold of 94.5% (Yarza et al., 2014). These results suggested that strain XS-1 represents a novel genus within the *Nitrospirota* MTB lineage. Here, we tentatively named it as *Candidatus* Magnetocorallium paracelense XS-1.

### 3.5. Central carbon metabolism and carbon fixation

**Figure 4:**
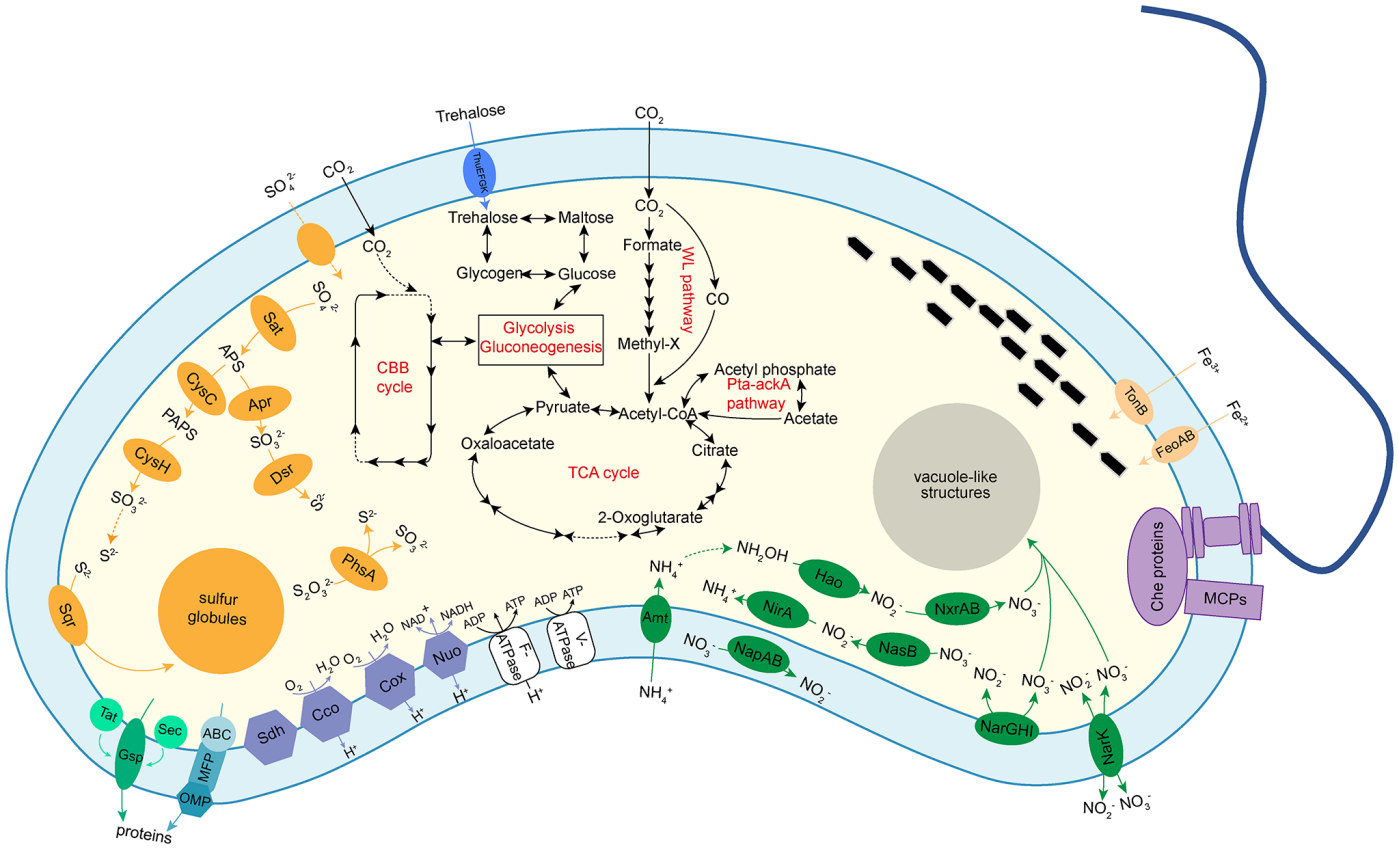
Diagrams of metabolic potential and electron transport of *Ca*. Magnetocorallium paracelense XS-1 based on gene content. Dotted arrows indicate that a pathway is partially complete. The following metabolic pathways were emphasized: glycolysis and gluconeogenesis; TCA cycle; autotrophic CO_2_ fixation through WL pathway and acetate fermentation through Pta-ackA pathway; trehalose and glycogen biosynthesis and degradation pathways; dissimilatory and assimilatory sulfite reductase pathways; dissimilatory and assimilatory nitrate reduction pathways; chemotaxis complex was represented by methyl-accepting chemotaxis proteins (MCPs) and Che proteins; a single polar flagellum; and the oxidation phosphorylation complexes and secretion systems.

The genome of XS-1 contains the complete gene repertoire for the glycolysis and gluconeogenesis pathways. It also encodes the almost complete tricarboxylic acid (TCA) cycle and the non-oxidative phase of the pentose phosphate pathway (Figure 4; Supplementary Table S3). Because of the absence of succinyl-CoA synthetase, the incomplete TCA cycle of XS-1 potentially serves only anabolic rather than energetic functions.

All required genes for glycogen biosynthesis via ADP-glucose, and the gene encoding glycogen phosphorylase (DJD_03816), which is responsible for the degradation of glycogen, are present in the XS-1 genome (Supplementary Figure S3A). Furthermore, *treSZY* genes involved in trehalose biosynthesis are identified as a compact gene cluster in the genome. The fact that genes encoding enzymes for trehalose degradation (trehalase, trehalose phosphorylase, and trehalose-6-phosphate hydrolase) are absent from the genome suggests that the principal role of trehalose in strain XS-1 is as a stress metabolite (Vanaporn and Titball, 2020) rather than a carbon source. The genome also contains genes encoding Acetyl-CoA synthetase, acetate kinase, and phosphate acetyltransferase. These enzymes are coupled to constitute a phosphate acetyltransferase-acetate kinase pathway (Pta-ackA pathway), which is responsible for acetate utilization in some heterotrophic bacteria (He et al., 2016).

All of the genes for carbon fixation via the WL pathway are identified in the genome of XS-1 (Supplementary Figure S3A), while genes encoding key enzymes (citryl-CoA lyase and citrate-CoA ligase) in the reductive tricarboxylic acid (rTCA) cycle are absent. The genome of XS-1 encodes the putative IV-type ribulose1,5-bisphosphate carboxylase/oxygenase (RubisCO; DJD_03546), which is a common feature among all identified *Nitrospirota* MTB genomes (Jogler et al., 2010; Lin et al., 2014; Zhang et al., 2021). However, as reported in previous studies, this protein does not contain the carboxylase and oxygenase activity domains, and has been proposed to function in sulfur metabolism rather than carbon fixation (Jogler et al., 2010). Intriguingly, in addition to the RubisCO-like protein (IV type), the XS-1 genome also encodes phosphoribulokinase (Prk), the other key enzyme of the Calvin–Benson–Bassham (CBB) cycle; this differs from other *Nitrospirota* MTB (Supplementary Figure S3A). A lower GC content of the *prk* gene (35.30%) in XS-1 than in the whole genome (41.25%) suggests that XS-1 may acquire the *prk* gene horizontally.

### 3.6. Nitrogen metabolism

Assimilatory nitrate reductase for reducing nitrate to nitrite is encoded in the XS-1 genome, suggesting that XS-1 potentially uses the assimilatory nitrate reduction (ANR) pathway to utilize nitrate as a nitrogen source (Figure 4). In addition, a gene cluster encoding subunits of the membrane-bound nitrate reductases (NarGHI) is identified; this is responsible for the dissimilatory nitrate reduction (DNR) pathway for the reduction of nitrate to nitrite, and release of protons into the cell periplasm. There are three copies of the ammonium transporter gene in the XS-1 genome. Three copies of the hydroxylamine dehydrogenase encoding the *hao* gene are also detected, but ammonia monooxygenase encoding genes (*amo*) are absent, suggesting an incomplete nitrification pathway initiating from hydroxylamine (Supplementary Table S3). In addition, three copies of the *hcp* gene encoding hydroxylamine reductase are detected in the XS-1 genome; these are likely to be involved in hydroxylamine reduction and detoxification (Wolfe et al., 2002).

### 3.7. Sulfur metabolism

Strain XS-1 has the potential to convert ambient sulfate into sulfide via the assimilatory sulfur reduction (ASR) pathway (Figure 4), which is used to produce the fundamental building blocks of cells, including amino acids. Consistent with most of the *Nitrospirota* MTB genomes, the XS-1 genome encodes the core enzymes for dissimilatory sulfur reduction (DSR), including ATP sulfurylase (Sat), adenylyl-sulfate reductase (AprAB), and dissimilatory sulfite reductase (DsrAB and DsrC). All these enzymes could operate in reverse, mediating the oxidation of sulfide, sulfur, and sulfite to conserve energy for cell growth (Zhou et al., 2011; Grein et al., 2013; Muller et al., 2015). Furthermore, the XS-1 genome contains an *asrABC* gene cluster encoding anaerobic sulfite reductase (AsrABC), which has been reported extensively in *Salmonella typhimurium*. Previous studies have reported that AsrC shares a conserved ferredoxin domain with DsrA and DsrB, indicating that AsrC is potentially involved in dissimilatory sulfite reduction (Dhillon et al., 2005). The XS-1 genome contains two copies of coding genes of the thiosulfate reductase subunit PhsA, which mediates the initial step in the disproportionation of thiosulfate, producing sulfite and sulfide (Supplementary Figure S3C). Another protein encoded by XS-1 genome, sulfide:quinone oxidoreductase (Sqr), participates in the oxidation of sulfide to sulfur and the formation of sulfur globules. A gene cluster in the XS-1 genome, *soeABC*, encodes the subunits of sulfite quinone dehydrogenase, which catalyzes the oxidation of sulfite to sulfate in the cell cytoplasm.

### 3.8. Oxidative phosphorylation

**Figure 5:**
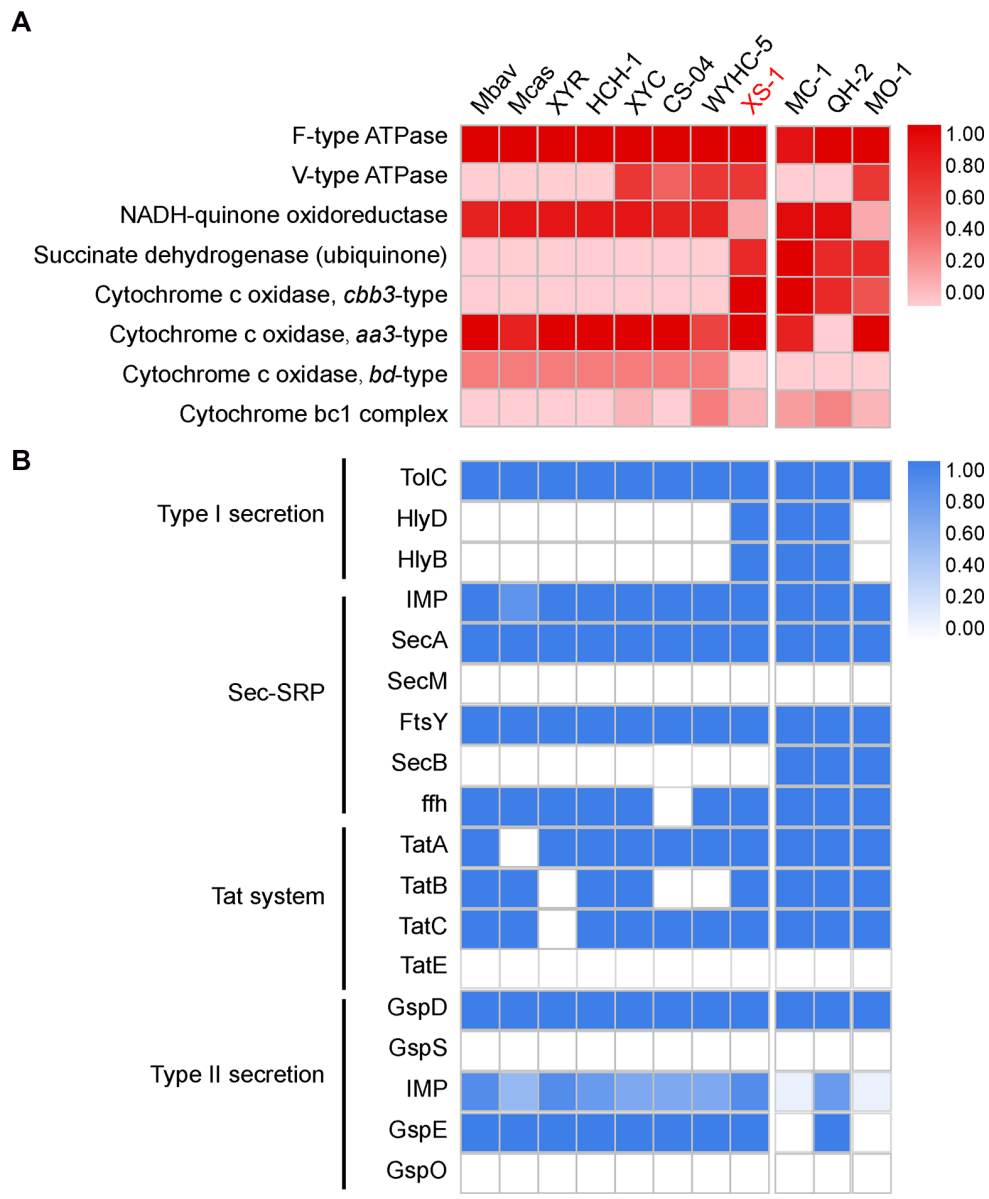
Oxidative phosphorylation and secretion systems of XS-1, and comparison with representative *Nitrospirota* MTB and marine MTB. **(A)** The heatmap shows the completeness of oxidative phosphorylation complexes in XS-1 and reference genomes. **(B)** The heatmap shows the completeness of secretory system components in XS-1 and reference genomes. Mbav: *Candidatus* Magnetobacterium bavaricum; Mcas: *Candidatus* Magnetobacterium casensis; XYR: *Candidatus* Magnetobacterium cryptolimnobacter XYR; HCH-1: *Candidatus* Magnetominusculus xianensis HCH-1; XYC: *Candidatus* Magnetomicrobium cryptolimnococcus XYC; CS-04: *Candidatus* Magnetoovum chiemensis CS-04; WYHC-5: *Candidatus* Magnetoovum sp. WYHC-5; MC-1: *Magnetococcus* marinus strain MC-1; QH-2: *Magnetospira* sp. QH-2; MO-1: *Magnetococcus* massalia strain MO-1.

The XS-1 genome contains two types of terminal cytochrome c oxidase, the *aa_3_*-type and the *cbb_3_*-type (Figure 5A). The *aa_3_*-type cytochrome c oxidase encoded by the gene cluster *coxABCD* and *cyoE* has a low affinity for oxygen, and functions best under high oxygen conditions. With respect to the *cbb_3_*-type cytochrome c oxidase, in the XS-1 genome there is one copy each of the genes for subunit I (CcoN), subunit II (CcoO), and subunit III (CcoP), but not for subunit IV (CcoQ); there is an additional copy of the *ccoP* gene on the same contig. In contrast to the *aa_3_*-type oxidase, the *cbb_3_*-type oxidase is a high affinity terminal oxidase that has been reported to be used by anaerobes and microaerophiles in microaerobic energy metabolism. In addition, the XS-1 genome contains a complete succinate dehydrogenase (SdhABC) which is absent from the genome of other freshwater *Nitrospirota* MTB (Figure 5A). This is also the case in some marine MTB from various lineages, suggesting that this feature is largely a consequence of environmental selection.

### 3.9. Secretion system

The XS-1 genome contains the genes for the components of the type I secretion system (T1SS), including the outer membrane TolC component, the periplasmic membrane fusion protein (MFP), and the inner membrane component (IMC). Genome comparison shows that the genomes of three marine MTB (MC-1, HQ-2, and XS-1) contain the complete T1SS. In contrast, the representative freshwater *Nitrospirota* MTB only contain the outer membrane TolC component (Figure 5B). Genomic information also reveals that XS-1 contains the transmembrane channel SecYEG, ATPase motor SecA, and some precursor proteins involved in the general secretory protein transport system (Sec system). The Sec system can transport unfolded proteins across the inner membrane and integrate α-helical proteins into the inner membrane. Genes for TatAc, TatB, and TatC are also detected in the XS-1 genome; these are involved in the Tat system responsible for transporting folded proteins across the inner membrane. The XS-1 genome also encodes some of the general secretion proteins (GPS) of the type II secretion system (T2SS; Supplementary Table S3), which is involved in the transport of periplasmic substrates across the outer membrane into the extracellular environment.

### 3.10. Iron metabolism and magnetosome biomineralization

**Figure 6:**
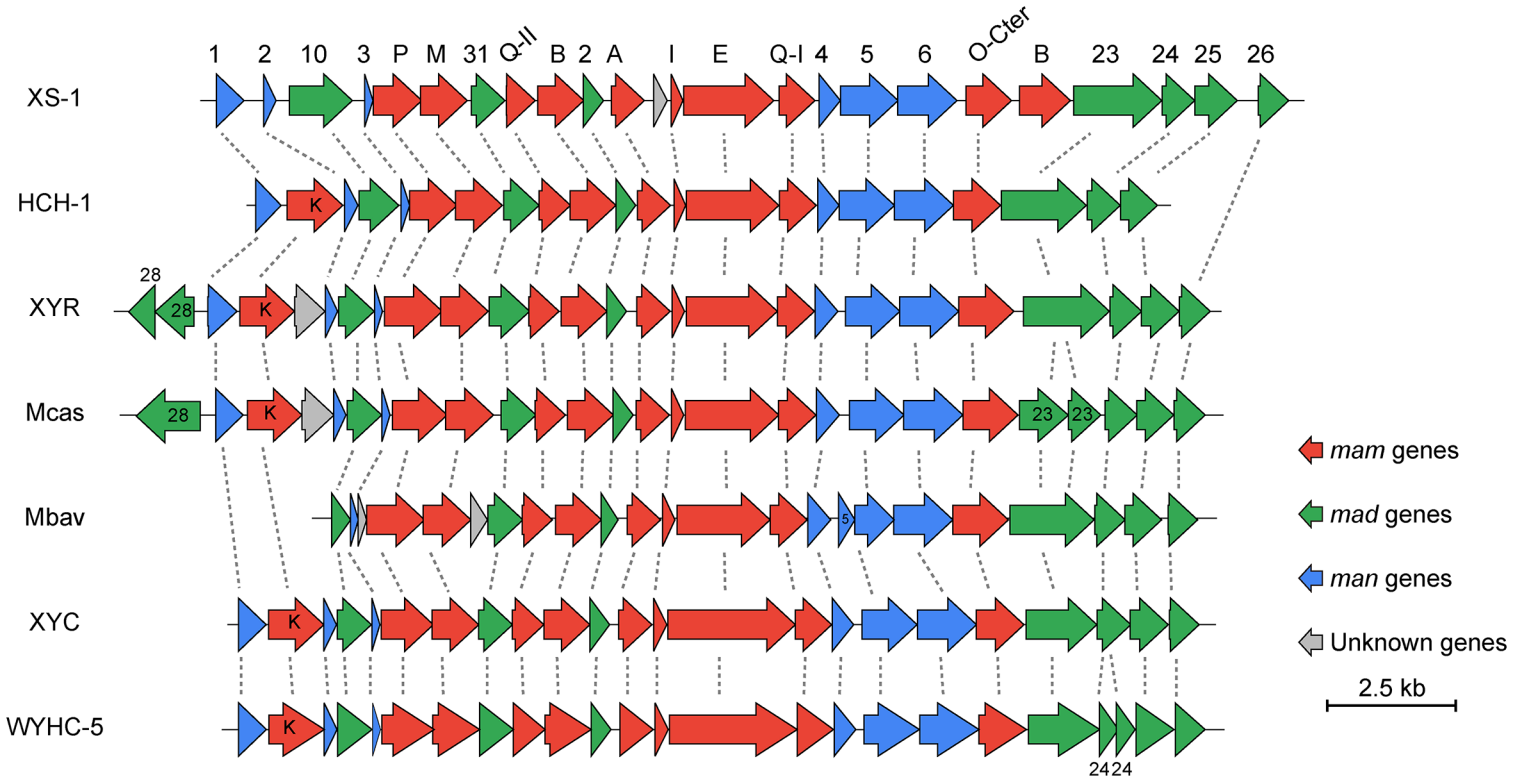
Comparison of MGCs from XS-1 and representative *Nitrospirota* MTB. The lines between clusters indicate homologous genes. Arrows show the genes and their transcriptional direction. HCH-1: *Candidatus* Magnetominusculus xianensis HCH-1; XYR: *Candidatus* Magnetobacterium cryptolimnobacter XYR; Mbav: *Candidatus* Magnetobacterium bavaricum; XYC: *Candidatus* Magnetomicrobium cryptolimnococcus XYC; WYHC-5: *Candidatus* Magnetoovum sp. WYHC-5.

Most of the genes for bullet-shaped magnetosome synthesis are detected in the reconstructed XS-1 genome. The main conserved magnetosome gene cluster (MGC) region of XS-1 (approximately 20 kb) is composed of 24 genes, 23 of which are homologous to known magnetosome genes (Figure 6; Supplementary Table S3). The *man1*-*mad26* gene cluster are considered to have the conserved gene content and gene order among *Nitrospirota* MTB (Zhang et al., 2021). There are several differences in gene content and the arrangement of the MGC in XS-1. Firstly, the core gene *mamK*, encoding cytoskeleton-like proteins for anchoring magnetosomes, is absent from the MGC, and an additional copy of the *mamB* gene, thought to be responsible for iron transport and magnetosome vesicle formation in MSR-1 (a model strain of *Proteobacteria* MTB; Uebe et al., 2018), is found located between *mamO-Cter* and *mad23*. Lastly, there is a gene of unknown function located between *mamA* and *mamI* (Figure 6), which has no homologous gene in known MTB. According to the NCBI Conserved Domain Database (Lu et al., 2020), this protein contains a four-helix bundle domain which is a ubiquitous structural motif in nature. In addition to this gene cluster, there is a small magnetosome-related gene cluster located on another contig, including *mad30*, *mad17*, *mad28*, and an unknown gene (Supplementary Table S3). Mad17 and Mad30 are likely to be involved in ferrous iron uptake for magnetosomes biomineralization, while Mad28 is a member of the actin family, participating in the positioning and assembly of magnetosome chains in δ-proteobacteria MTB (Lefèvre et al., 2013).

### 3.11. Circadian rhythm

The genome of XS-1 contains four copies of the *kaiBC* gene cluster and an additional uncoupled copy each of *kaiB* and *kaiC* (Supplementary Table S3). The amino acid identities shared by the five KaiB proteins range from 31.08 to 51.81%, while for the five KaiC copies the range is 32.93 to 66.40%. Three of the *kaiBC* gene clusters are located on the same contig, and are separated by 15 genes. Most of these genes encode proteins with histidine kinase domains, which may act as sensors for environmental stimuli of signal transduction (Supplementary Table S3). KaiBC is the core component of the *kaiBC*-based clock protein complex. The existence of a circadian clock has been demonstrated for some prokaryotes, including *Cyanobacteria* and *Archaea* (Iwasaki and Kondo, 2004; Schmelling et al., 2017). A common feature among some species is the presence of multiple copies of *kai* genes; for example, *Synechocystis* sp. PCC6803 has three copies of *kaiB* and *kaiC*. MTB seem to be a represent of the multicopy members. Several copies of *kaiBC* have been described, respectively, in three MMP genomes (Abreu et al., 2014; Kolinko et al., 2014; Qian et al., 2019b), and we also found *kaiBC* of two copies in some freshwater *Nitrospirota* MTB (Supplementary Table S4). Intriguingly, among all these MTB, XS-1 has the largest number of gene copies of *kaiB* and *kaiC*.

**Figure 7:**
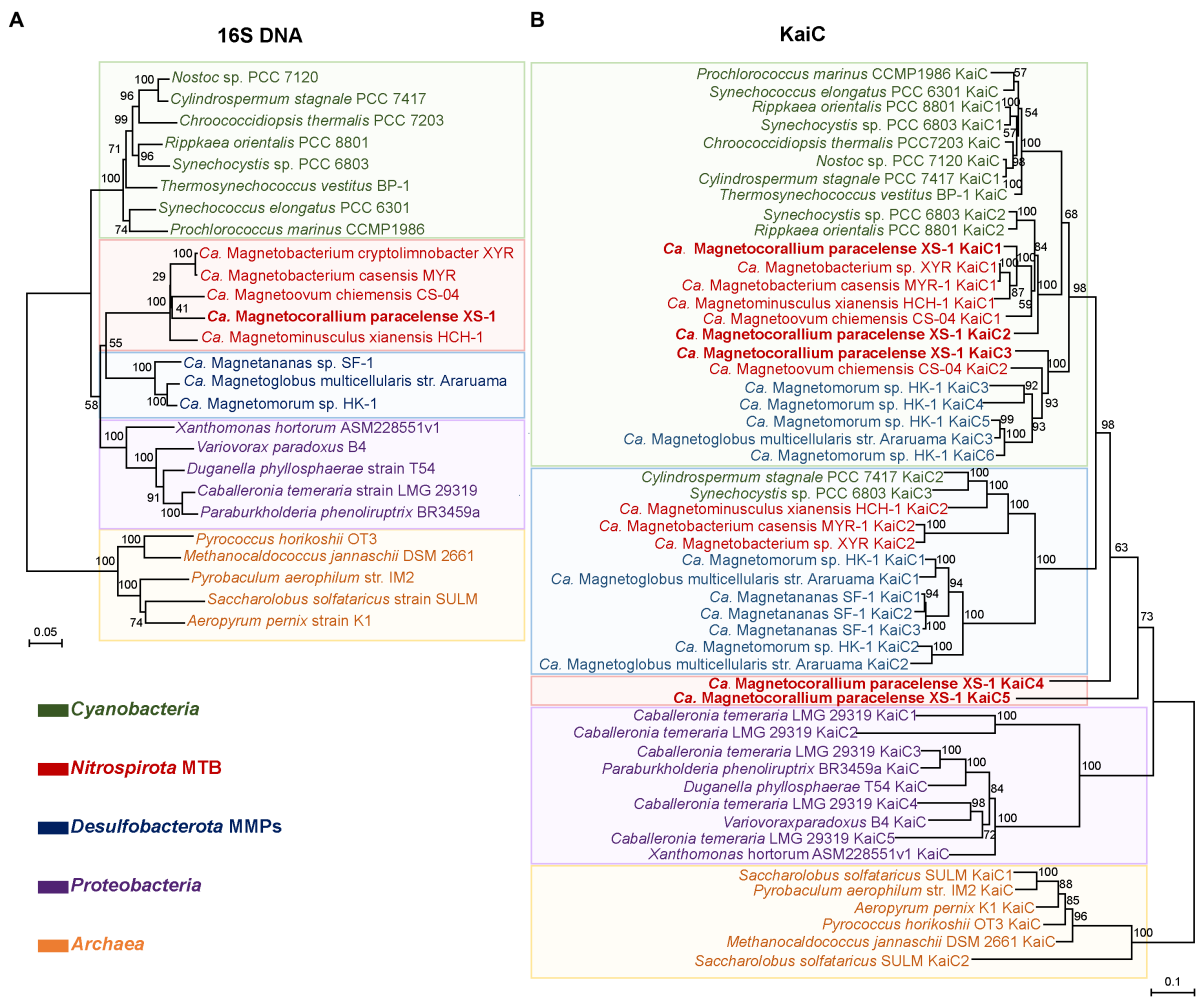
Comparison of 16S rDNA- and KaiC-based phylogenetic consensus trees. The 16S rRNA genes **(A)** and KaiC proteins **(B)** were used for neighbor-joining and maximum likelihood phylogenetic reconstruction. Bootstrap values at nodes were given as percentages of 1,000 replicates. Sequences from this study are marked in bold. The shaded colors divide each of the two phylogenetic trees into five major clades, and the color is determined by the dominant taxon of the clades.

A KaiC-based phylogenetic tree of the major rhythm-containing lineages and a 16S rDNA-based tree of the same set of organisms was constructed and compared to gain insights into the evolution of KaiC (Figure 7; Supplementary Table S4). The evolution pattern of the major copy of KaiC among the lineages is broadly in accordance with the species evolution. For *Nitrospirota* MTB, the extra copies cluster approximately into three subclades on the KaiC-based tree. The major subclade containing most *Nitrospirota* MTB sequences form a larger lineage with the *Cyanobacteria* clade (Figure 7, green box). Three copies of XS-1 sequences join into this clade. Another subclade contains extra copies of three freshwater *Nitrospirota* MTB (*Ca.* Magnetobacterium casensis, *Ca.* Magnetominusculus xianensis, and *Ca.* Magnetobacterium sp. XYR), which joins into the *Desulfobacterota* clade together with two sequences from *Cyanobacteria* (Figure 7, bule box). The third *Nitrospirota* subclade is consisted of the other two copies from XS-1, which seems to form an outgroup of the above lineages, but support for the node was relatively low (68%; Figure 7). These findings suggest that the multiple KaiC-gene copies in XS-1, or even in the entire *Nitrospirota* MTB lineage, are results of multiple evolutionary events, including gene duplication, mutation, and horizontal gene transfer.

## 4. Discussion

Marine *Nitrospirota* MTB were found most recently in a lagoon (Six-Four les Plages) of the Mediterranean Sea (Qian et al., 2019a). They were considered to play important roles in marine biogeochemical cycles due to their outstanding ability to biomineralize large amounts of magnetite magnetosomes and sulfur globules. However, few studies have been carried out investigating their detailed metabolic potential and ecological functions. In this study we identified a novel *Nitrospirota* MTB (*Candidatus* Magnetocorallium paracelense XS-1) obtained from the coral reef area of the Xisha Islands (South China Sea), and provide here its genome sequence, the first such report for a marine *Nitrospirota* MTB. Strain XS-1 is remarkable for its phylogenetic position, forming a distinct branch distant from all known *Nitrospirota* species on the phylogenomic tree, so it was not surprising to find that XS-1 has novel morphology and genetic characteristics. An intuitive characteristic was the low number of magnetosomes in XS-1 cells compared with most of the *Nitrospirota* MTB from freshwater (Lin et al., 2011, 2012, 2014; Lefèvre et al., 2011a,b; Qian et al., 2019a; Zhang et al., 2021), and MMPs from the same coral reef area (Teng et al., 2018b). The bullet-shaped magnetosomes of XS-1 cells are arranged in bundles, but more loosely than in other *Nitrospirota* MTB. Possible reasons can be inferred from the fact that the highly conserved gene content and order of MGCs in freshwater *Nitrospirota* MTB are somewhat different from those in XS-1 (Zhang et al., 2021). The core gene *mamK*, which encodes the magnetosome-associated cytoskeleton protein, was missing from the MGCs of XS-1. Researchers have noted that the loss of *mamK* in an MSR-1 mutant strain results in short magnetosome chains and fewer magnetosomes (Komeili et al., 2006; Katzmann et al., 2010). Thus, the lack of *mamK* may explain, at least in part, the loose magnetosome chains and fewer magnetosomes in XS-1 cells.

XS-1 is the sole marine *Nitrospirota* MTB for which there is available genomic information, enabling comparison of its metabolism potential with that of freshwater *Nitrospirota* MTB. Similar to other *Nitrospirota* MTB (Lin et al., 2014; Kolinko et al., 2016; Zhang et al., 2021), XS-1 contains the WL pathway for CO_2_ fixation and the dissimilatory sulfur reduction (DSR) pathway for energy production, indicating a chemoautotrophic lifestyle. However, there were differences between the marine and freshwater species. Firstly, XS-1 contains a complete phosphate acetyltransferase-acetate kinase pathway (Pta-ackA pathway), which enables cells to convert acetyl-CoA to acetate (Enjalbert et al., 2017), and to compensate for energy consumption in the methyl branch of the WL pathway. Second, the XS-1 genome contains *arsABC* and *phsA* genes for anaerobic sulfite reduction and thiosulfate disproportionation, suggesting that XS-1 can potentially obtain energy through the disproportionation of sulfur or thiosulfate, and has more metabolic pathways involved in the sulfur cycle (Umezawa et al., 2020). In contrast to *Nitrospirota* bacteria, XS-1 is unable to perform nitrogen fixation, and all other nitrogen metabolism pathways in the MAG were incomplete, which suggests this strain cannot independently access inorganic nitrogen for growth, and may act cooperatively with other microbial communities to complete the nitrogen cycle. These features of XS-1 enable it to save energy and adapt to changing environments, so that increase its chance of survival in oligotrophic marine environments.

The major differences between XS-1 and freshwater *Nitrospirota* MTB lie in the details of the electron transport chain complexes. XS-1 has both the high oxygen affinity terminal oxygen reductases (cytochrome *cbb_3_*-type quinol complex; Pitcher and Watmough, 2004) and the low oxygen affinity terminal oxygen reductases (*aa_3_*-type cytochrome c oxidases; Arai et al., 2014), which are thought to provide survival advantages in the marine habitats where current and tide might create varying oxygen concentrations. It has been shown that the loss of the *cbb_3_*-type caused complex magnetosome phenotypes and aberrant cell morphologies in the *M. gryphiswaldense* mutant strain, whereas loss of the *bd*-type, another cytochrome c oxidase common in MTB, had no effect on growth or magnetosome synthesis (Li et al., 2014). On the other hand, incubation of a non-MTB strain, *Shewanella oneidensis* MR-1, under iron deficiency conditions resulted in a significant increase in the expression of *cbb_3_*-type cytochrome c oxidase (Le Laz et al., 2016). Consequently, the presence of *cbb_3_*-type cytochrome c oxidase is a strategy that enables XS-1 to better adapt to dissolved-oxygen fluctuating and iron-deficient marine environments, and also help to regulate the redox conditions for magnetosome biomineralization.

The local environmental constraints may have substantially shaped the cellular metabolism of XS-1 in the coral reef area it inhabits. One exciting finding in XS-1 genome is the identification of the highest numbers of gene copies encoding the *kaiBC*-based circadian rhythm systems. Circadian rhythm systems have been classified into three form classes, depending on the protein components of the circadian clock complex: the *kaiABC*-based system, the *kaiBC*-based system, and the *kaiC*-based system. The *kaiABC*-based and *kaiC*-based system was commonly found in *Cyanobacteria* (Iwasaki and Kondo, 2004) and halophilic archaea (Maniscalco et al., 2014), respectively. The *kaiBC*-based system has been found in species of *Proteobacteria* (Ma et al., 2016), the cyanobacterial genus *Prochlorococcus* (Holtzendorff et al., 2008), and now in *Nitrospirota* MTB. The *kaiBC*-based circadian rhythm system was proposed to be a timekeeping mechanism, and has an important effect on growth and fitness of purple non-sulfur bacteria strains (Ma et al., 2016). Abreu et al. (2014) and Qian et al. (2019b) have reported the multiple copies of *kaiBC* in the genome of MMPs *Ca.* Magnetoglobus multicellularis str. Araruama and *Ca.* Magnetananas sp. SF-1, respectively. In particular, another MMP species *Ca.* Magnetomorum sp. HK-1 was found to have six copies of each of the two genes in its MAG (Qian et al., 2019b). These organisms have the most complex life cycle and natural physiological behavior among MTB. The *kaiBC*-based circadian rhythm system is considered to help them to adapt to circadian variation of chemical gradients and light intensity. Given that coral reefs are the most dynamic marine systems and are subject to substantial fluctuations in nutrient levels, oxygen/CO_2_ levels, and diurnal temperature (Weber and Apprill, 2020), it is not surprising that XS-1 identified in coral reef sediments contains extra copies of circadian rhythm genes to adaptively match their environment.

**Figure 8:**
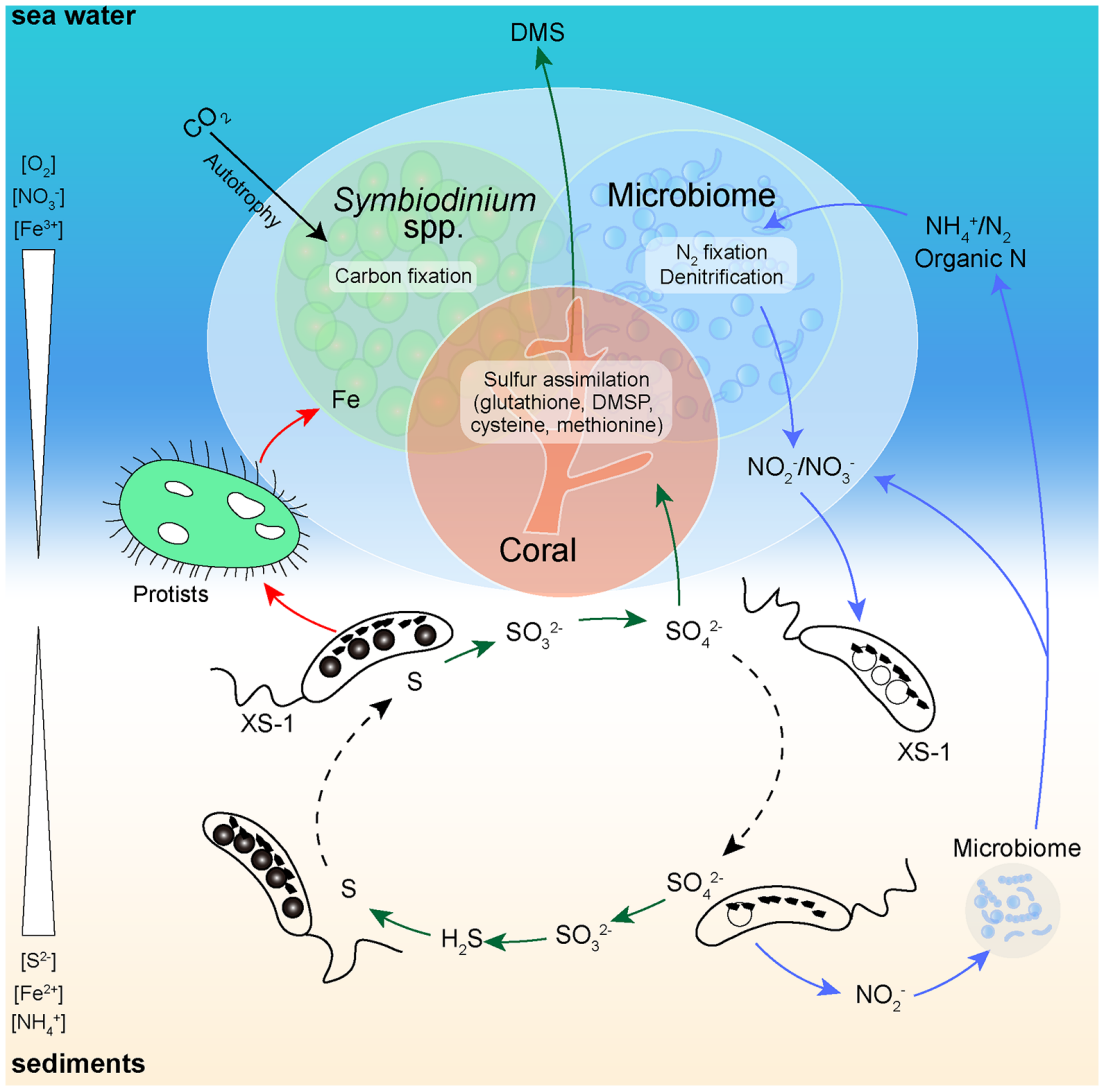
Simplified schematic diagram of the known functional roles of XS-1 in coral sediments. At the micro-oxic zone of coral reef sediments, XS-1 cells carry out sulfur oxidation, accumulate nitrate (forming vacuole-like structures), and release sulfate and bioavailable iron into the environment. The green arrows show the flow of sulfur. The red arrows show the flow of iron. The blue arrows show the flow of nitrogen. The black arrow shows the process of carbon fixation in the coral holobiont. The swimming directions of XS-1 are indicated with dashed black arrows.

The ecological function of XS-1 in coral reef ecosystems was also considered. XS-1 potentially has versatile metabolic pathways of sulfur which could benefit the coral reef health (Figure 8). Sulfide is generally released into the environment by sulfate-reducing bacteria, which are widely distributed (Kushkevych et al., 2021), and coral reef habitats are also subject to the potential occurrence of hydrogen sulfide toxicity. Because of its sulfide oxidation capacity, XS-1 may contribute to the removal of harmful H_2_S from coral reef sediments. XS-1 also encodes enzymes for the reverse dissimilatory sulfate reductase pathway, which oxidizes stored intracellular sulfur to sulfate via sulfite. This provides soluble sulfate for the synthesis of dimethylsulfoniopropionate (DMSP), which is considered to function as a stress protectant for coral species (Raina et al., 2010, 2013). It should be noted that XS-1 may be in low initial abundance in the local sediments, which makes it essential as rare biosphere in ensuring the functional redundancy of the unstable coral reef ecosystems (Lynch and Neufeld, 2015). Coral reefs are habitats where the primary productivity largely depends on nitrogen availability. This highlights the importance of biogeochemical cycling of nitrogen in these environments (Ceh et al., 2013). We observed vacuole-like structures within XS-1 cells, which resemble the nitrate-storing vacuoles proposed in ecological studies of *Ca.* Magnetobacterium casensis (Li et al., 2020). Thus, XS-1 may play a role in some steps of the nitrogen metabolic network by retaining a nitrogen source pool for the environment. In addition, strain XS-1 exhibited directional motion that allow them to shuttle up and down through the sediments, thus enabling the microbial nitrogen cycling in coral reef ecosystems (Figure 8). On the other hand, MTB have been found to be a minor part of the microbial community in some deep sea and bog soil environments and are considered to have a special functional role in these ecosystems (Sogin et al., 2006; Zapata-Peñasco et al., 2018; Uzun et al., 2023). The most prominent feature of XS-1 is the large amounts of magnetosome produced by biomineralization in their cells. It has been estimated that the synthesis of magnetosomes in unicellular MTB varies from 2.6 to 41.7 mg/l, depending on the culture conditions (Araujo et al., 2015). Accordingly, MTB can accumulate up to 100-fold more iron than other non-MTB (Schüler and Baeuerlein, 1996). Statistical analysis in this study indicates that the average dry weight of iron per XS-1 cell is up to 2.30% of the cell biomass. XS-1 cells are capable to take up free iron from coral reef environments through the synthesis of magnetosomes, thus limiting overgrowth by phytoplankton and heterotrophic microorganisms that are likely to cause the formation of black reefs (Work et al., 2008; Kelly et al., 2012; Twining and Baines, 2013). In addition, MTB are common prey for protozoa, so bioavailable iron is likely to be released into the environment after the dissolution of magnetosomes by the protozoal native acidic vacuoles, following the predation of the MTB cells (Martins et al., 2007; Monteil et al., 2018; Chen et al., 2021). As shown in Figure 8, a schematic representation of coral-microbe interaction involving sediment microorganisms such as XS-1 can be found.

## 5. Conclusion

*Nitrospirota* MTB have never been found in the most important marine ecosystems including coral ecosystem. Our study provides a comprehensive analysis of a newly identified *Nitrospirota* MTB in tropical coral sediments. *Candidatus* Magnetocorallium paracelense XS-1 represents a novel genus of the phylum *Nitrospirota*, and is the first marine *Nitrospirota* MTB for which genome data are available. Genome comparisons demonstrated that XS-1 has somewhat different metabolic potential from previously described freshwater *Nitrospirota* MTB. When XS-1 cells shuttle through the oxic-anoxic transition zone of coral-associated sediments through magnetotaxis, they may drive the biogeochemical cycling of key elements including C, N, S, and Fe, and thus participate in coral ecological processes. To increase insights into the mechanisms of coral ecosystems and to aid ecological protection, future studies involving broader surveys of the bacterial populations in coral-associated sediments as well as focusing on special functional microbes should occur.

## Data availability statement

The datasets presented in this study can be found in online repositories. The names of the repository/repositories and accession number(s) can be found at: https://www.ncbi.nlm.nih.gov/, JAREWP000000000; https://www.ncbi.nlm.nih.gov/genbank/, OQ281288.

## Author contributions

TX, WZ, and JL designed the research. YZ, KC, and HP performed the experiment. YZ, WZ, and JC contributed to sequence data analysis and assembly. YZ and JL performed the genome data analyses and original draft preparation. All authors contributed to the article and approved the submitted version.

## Funding

This work was supported by research grants from the National Natural Science Foundation of China (grants no. NSFC 42206089 and NSFC 42176123).

## Conflict of interest

The authors declare that the research was conducted in the absence of any commercial or financial relationships that could be construed as a potential conflict of interest.

## Publisher’s note

All claims expressed in this article are solely those of the authors and do not necessarily represent those of their affiliated organizations, or those of the publisher, the editors and the reviewers. Any product that may be evaluated in this article, or claim that may be made by its manufacturer, is not guaranteed or endorsed by the publisher.

## Footnotes

^1^ https://blast.ncbi.nlm.nih.gov/Blast.cgi

^2^ https://github.com/dparks1134/CompareM

## Supplementary material

Supplementary Movie S1 The polar north-seeking motility of XS-1.

Supplementary Movie S2 The ‘volcano motility behavior’ of XS-1 occurring at the edge of the water droplet.

Supplementary Figure S1 Transmission electron micrographs of XS-1. **(A)** TEM images showing bullet-shaped magnetosomes loosely arranged in bundles roughly lying along the long cell axis. **(C)** TEM images showing bullet-shaped magnetosomes scattered in the XS-1 cell. **(B)** and **(D)** are the magnified images of the area marked by dash line rectangle in **(A)** and **(C)**, respectively. Red arrows indicate the immature magnetosomes.

Supplementary Figure S2 Neighbor-joining tree based on 16S rRNA gene sequences. The multiple sequence alignment consisting of 16S rRNA genes contained *Nitrospirota* MTB and non-MTB, rooted with representative members of the *Deferribacterota* phylum. The bootstrap support values (out of 1000 replicates) are indicated as percentages. The sequence in this study is highlighted in red.

Supplementary Figure S3 Carbon, nitrogen, and sulfur metabolism maps for XS-1, and comparisons with representative *Nitrospirota* MTB genomes. The maps show carbon **(A)**, nitrogen **(B)** and sulfur **(C)** metabolism based on the XS-1 genome, and the heatmaps show the presence of genes encoding the predicted pathway in XS-1 and other *Nitrospirota* MTB genomes. Mbav: *Candidatus* Magnetobacterium bavaricum; Mcas: *Candidatus* Magnetobacterium casensis; XYR: *Candidatus* Magnetobacterium cryptolimnobacter XYR; HCH-1: *Candidatus* Magnetominusculus xianensis HCH-1; XYC: *Candidatus* Magnetomicrobium cryptolimnococcus XYC; CS-04: *Candidatus* Magnetoovum chiemensis CS-04; WYHC-5: *Candidatus* Magnetoovum sp. WYHC-5.
