# Supplementary material for: Insight into the metabolic potential and ecological function of a novel Magnetotactic Nitrospirota in coral reef habitat
Source: Front Microbiol. 2023 May 17;14:1182330. doi: 10.3389/fmicb.2023.1182330 (PMC10278575; doi:10.3389/fmicb.2023.1182330)
Supplement: Supplementary file 1 [file Table_1.pdf]

Table S1 General genomic features of the draft genome of XS-1

Parameter	XS-1
Total genome size (Mb)	6.01
N50 (bp)	99 879
GC%	41.25
Number of contigs	103
Number of coding sequences (CDS)	4 858
Average contig length (bp)	1 034.37
5/16/23S rRNA	2001/1/1
tRNA	47
Completeness	97.27%
Contamination	3.64%

Table S2 The AAI matrix of *Nitrospirota* MTB

	XS-1	HCH-1	Mbav	Mcas	XYR	CS04	XYC	WYHC-5
XS-1	——							
HCH-1	57.25%	——						
Mbav	55.74%	60.10%	——					
Mcas	56.89%	61.03%	83.52%	——				
XYR	56.50%	60.63%	83.69%	92.52%	——			
CS04	56.21%	57.02%	56.23%	57.34%	56.74%	——		
XYC	56.11%	57.51%	56.89%	57.50%	57.24%	58.53%	——	
WYHC-5	50.90%	53.69%	53.06%	53.80%	53.76%	55.40%	99.63%	——

Table S3 List of genes involved in the major metabolic pathways

Metabolism involvement	Contig	Gene ID	Gene name	Product
**Carbon**	k255_10025	DJD_04718	*cooS*	carbon-monoxide dehydrogenase, catalytic subunit
	k255_6150	DJD_01895	*cooS*	carbon-monoxide dehydrogenase, catalytic subunit
	k255_10025	DJD_04722	*fdhA*	formate dehydrogenase (hydrogenase)
	k255_46175	DJD_01564	*fhs*	Formate--tetrahydrofolate ligase
	k255_24565	DJD_00810	*folD*	Methenyltetrahydrofolate cyclohydrolase / Methylenetetrahydrofolate dehydrogenase (NADP+)
	k22_61170	DJD_02827	*folD*	Methenyltetrahydrofolate cyclohydrolase / Methylenetetrahydrofolate dehydrogenase (NADP+)
	k255_61170	DJD_02825	*metF*	5,10-methylenetetrahydrofolate reductase
	k255_41725	DJD_00065	*acsE*	5-methyltetrahydrofolate corrinoid/iron sulfur protein methyltransferase
	k255_41725	DJD_00067	*cdhE*	Acetyl-CoA synthase corrinoid iron-sulfur protein, large subunit
	k255_41725	DJD_00066	*cdhD*	Acetyl-CoA synthase corrinoid iron-sulfur protein, small subunit
	k255_10025	DJD_04717	*acsB*	CO dehydrogenase/acetyl-CoA synthase, acetyl-CoA synthase subunit
	k255_57676	DJD_01840	*pta*	Phosphate acetyltransferase
	k255_72157	DJD_01602	*ackA*	acetate kinase
	k255_12352	DJD_03149	*Acs*	Acetyl-CoA synthetase
	k255_58366	DJD_04242	*prk*	phosphoribulokinase
	k255_10952	DJD_03546	*rbcL*	Ribulose 1,5-bisphosphate carboxylase, large subunit, or a RuBisCO-like protein
	k255_36877	DJD_04130	*pgk*	phosphoglycerate kinase
	k255_8715	DJD_01146	*gapA*	glyceraldehyde 3-phosphate dehydrogenase (phosphorylating)
	k255_36877	DJD_04129	*gapA*	glyceraldehyde 3-phosphate dehydrogenase (phosphorylating)
	k255_58849	DJD_04758	*fba*	fructose-bisphosphate aldolase, class II
	k255_34859	DJD_02576	*fbp*	fructose-1,6-bisphosphatase I
	k255_30799	DJD_02495	*tktA*	transketolase
	k255_14910	DJD_03084	*tktA*	transketolase
	k255_30799	DJD_02496	*tktA*	transketolase
	k255_14910	DJD_03085	*tktA*	transketolase
	k255_10952	DJD_03580	*tktA*	transketolase
	k255_61013	DJD_02325	*rpiB*	ribose 5-phosphate isomerase B
	k255_73342	DJD_00323	*gltA*	Citrate Synthase

k255_32309	DJD_03383	*canA*	Aconitate hydratase
k255_61013	DJD_02326	*idh*	Isocitrate dehydrogenase [AND$^+$]
k255_58849	DJD_04836	*korA*	2-oxoglutarate/2-oxoacid ferredoxin oxidoreductase subunit alpha
k255_47382	DJD_04657	*korB*	2-oxoglutarate/2-oxoacid ferredoxin oxidoreductase subunit beta
k255_10952	DJD_03623	*korD*	2-oxoglutarate ferredoxin oxidoreductase subunit delta
k255_47382	DJD_04658	*korC*	2-oxoglutarate ferredoxin oxidoreductase subunit gamma
k255_7275	DJD_00435	*sdhA*	fumarate reductase flavoprotein subunit
k255_45298	DJD_02775	*sdhB*	succinate dehydrogenase iron-sulfur subunit
k255_9435	DJD_03853	*sdhC*	succinate dehydrogenase / fumarate reductase, cytochrome b subunit
k255_33169	DJD_01349	*fumA*	fumarate hydratase, class I
k255_10025	DJD_04723	*fumA*	Fumarate hydratase class I
k255_23130	DJD_00949	*mdh*	malate dehydrogenase
k255_41778	DJD_00746	*glgC*	glucose-1-phosphate adenylyltransferase
k255_30799	DJD_02477	*glgA*	starch synthase
k255_48994	DJD_00466	*glgB*	1,4-alpha-glucan branching enzyme
k255_45298	DJD_02762	*glgB*	1,4-alpha-glucan branching enzyme
k255_12637	DJD_03816	*malQ*	glycogen phosphorylase
k255_58849	DJD_04878	*treX*	isoamylase
k255_34859	DJD_02614	*treY*	malto-oligosyltrehalose synthase
k255_34859	DJD_02613	*treZ*	maltooligosyltrehalose trehalohydrolase
k255_34859	DJD_02612	*treS*	maltose alpha-D-glucosyltransferase / alpha-amylase
k255_33169	DJD_01442	*malZ*	alpha-glucosidase
k255_9435	DJD_03857	*malZ*	alpha-glucosidase
k255_58849	DJD_04877	*malQ*	4-alpha-glucanotransferase
k255_23130	DJD_00945	*ppdk*	pyruvate, orthophosphate dikinase
k255_41604	DJD_04589	*ppdk*	pyruvate, orthophosphate dikinase
k255_58849	DJD_04846	*ppdk*	pyruvate, orthophosphate dikinase
k255_17323	DJD_00624	*ppsA*	pyruvate, water dikinase
k255_15989	DJD_00575	*eno*	Enolase
k255_14910	DJD_03057	*gpmI*	2,3-bisphosphoglycerate-independent phosphoglycerate mutase

	k255_20157	DJD_00183	*gpmM*	2,4-bisphosphoglycerate-independent phosphoglycerate mutase
	k255_39923	DJD_04633	*tpi*	triosephosphate isomerase (TIM)
	k255_41778	DJD_00694	*gpi*	glucose-6-phosphate isomerase
	k255_14910	DJD_03028	*glk*	Glucokinase
	k255_10952	DJD_03512	*rnfC*	Electron transport complex protein RnfC
	k255_10952	DJD_03513	*rnfD*	Electron transport complex protein RnfD
	k255_10952	DJD_03514	*rnfG*	Electron transport complex protein RnfG
	k255_10952	DJD_03515	*rnfE*	Electron transport complex protein RnfE
	k255_10952	DJD_03516	*rnfA*	Electron transport complex protein RnfA
	k255_10952	DJD_03517	*rnfB*	Electron transport complex protein RnfB
**Nitrogen**	k255_46175	DJD_01570	*narG*	nitrate reductase / nitrite oxidoreductase, alpha subunit
	k255_46175	DJD_01571	*narH*	nitrate reductase / nitrite oxidoreductase, beta subunit
	k255_46175	DJD_01572	*narI*	nitrate reductase gamma subunit
	k255_9435	DJD_03893	*napA*	Nitrate reductases, NapA (Nitrate-R-NapA)
	k255_61891	DJD_00488	*narB*	ferredoxin-nitrate reductase
	k255_17323	DJD_00639	*hao*	hydroxylamine oxidase
	k255_46175	DJD_01550	*hao*	hydroxylamine oxidase
	k255_10952	DJD_03523	*hao*	hydroxylamine oxidase
	k255_10025	DJD_04718	*hcp*	hydroxylamine reductase
	k255_32309	DJD_03232	*hcp*	hydroxylamine reductase
	k255_6150	DJD_01895	*hcp*	hydroxylamine reductase
	k255_12352	DJD_03127	*gltB*	glutamate synthase (NADPH) large chain
	k255_59323	DJD_02121	*gltD*	glutamate synthase (NADPH) small chain
	k255_13259	DJD_01954	*glnA*	glutamine synthetase
	k255_46175	DJD_01568	*narK*	Nitrate/nitrite transporter NarK
	k255_33169	DJD_01412	*amt*	ammonium transporter
	k255_13259	DJD_01952	*amt*	ammonium transporter
	k255_23029	DJD_00221	*amt*	ammonium transporter
**Sulfur**	k255_36877	DJD_04141	*sat*	sulfate adenylyltransferase
	k255_61170	DJD_02833	*cysN*	sulfate adenylytransfarase subunit 1

k255_61170	DJD_02834	*cysD*	sulfate adenylyltransferase, small subunit
k255_16252	DJD_01728	*cysC*	3'(2'),5'-bisphosphate nucleotidase
k255_36877	DJD_04139	*aprA*	Adenylylsulfate reductase alpha-subunit
k255_36877	DJD_04140	*aprB*	Adenylylsulfate reductase beta-subunit
k255_33169	DJD_01405	*dsrA*	Dissimilatory sulfite reductase, alpha subunit
k255_33169	DJD_01406	*dsrB*	Dissimilatory sulfite reductase, beta subunit
k255_34859	DJD_02618	*dsrC*	Sulfur redox associated protein DsrC
k255_46175	DJD_01573	*asrA*	anaerobic sulfite reductase subunit A
k255_46175	DJD_01574	*asrB*	anaerobic sulfite reductase subunit B
k255_46175	DJD_01575	*asrC*	anaerobic sulfite reductase subunit C
k255_34859	DJD_02649	*soeA*	sulfite dehydrogenase (quinone) subunit SoeA
k255_34859	DJD_02650	*soeB*	sulfite dehydrogenase (quinone) subunit SoeB
k255_34859	DJD_02651	*soeC*	sulfite dehydrogenase (quinone) subunit SoeC
k255_57521	DJD_00140	*sqr*	sulfide:quinone oxidoreductase
k255_33169	DJD_01202	*phsA*	thiosulfate reductase PhsA
k255_72157	DJD_01599	*phsA*	thiosulfate reductase PhsA
k255_24565	DJD_00834	*psrB*	polysulfide reductase beta (PsrB) subunit
k255_28324	DJD_04012	*cysK*	Cysteine synthase
k255_28324	DJD_04013	*cysM*	Cysteine sythase B
k255_32309	DJD_03255	*cysM*	Cysteine sythase B
k255_32309	DJD_03266	*sul*	sulfate permease
k255_70165	DJD_04170	*nrnA*	bifunctional oligoribonuclease and PAP phosphatase NrnA
k255_16252	DJD_01729	*cysQ*	3'(2'),5'-bisphosphate nucleotidase
k255_28324	DJD_04012	*cysK*	cysteine synthase
k255_17323	DJD_00598	*ssuA*	sulfonate transport system substrate-binding protein
k255_19956	DJD_03829	*tauA*	Taurine ABC transporter, substrate-binding protein TauA
k255_19956	DJD_03830	*tuaB*	Taurine ABC transporter, substrate-binding protein TauB
k255_19956	DJD_03831	*tuaC*	Taurine ABC transporter, substrate-binding protein TauC
k255_36877	DJD_04136	*qmoC*	Anaerobic respiratory complex protein QmoC
k255_36877	DJD_04137	*qmoB*	Anaerobic respiratory complex protein QmoB

	k255_36877	DJD_04138	*qmoA*	Anaerobic respiratory complex protein QmoA
**NADH dehydrogenase**	k255_10025	DJD_04719	*nuoE*	NAD(P)H-dependent oxidoreductase subunit E
	k255_33169	DJD_01392	*nuoM*	NADH-ubiquinone oxidoreductase chain M
	k255_33169	DJD_01393	*nuoM*	NADH-ubiquinone oxidoreductase chain M
	k255_33169	DJD_01396	*nuoM*	NADH-ubiquinone oxidoreductase chain M
	k255_61891	DJD_00490	*nuoF*	NAD(P)H-dependent oxidoreductase subunit F
**Succinate dehydrogenase (ubiquinone)**	k255_7275	DJD_00435	*sdhA*	succinate dehydrogenase, flavoprotein subunit
	k255_45298	DJD_02775	*sdhB*	succinate dehydrogenase, iron-sulfur subunit
	k255_9435	DJD_03853	*sdhC*	succinate dehydrogenase, cytochrome b subunit
**Cytochrome c oxidase, *aa3* -type**	k255_10025	DJD_04736	*coxC*	cytochrome c oxidase subunit III
	k255_10025	DJD_04737	*coxD*	cytochrome c oxidase subunit IV
	k255_10025	DJD_04738	*coxB*	cytochrome c oxidase subunit II
	k255_10025	DJD_04739	*coxA*	cytochrome c oxidase subunit I
	k255_10025	DJD_04740	*cyoE*	heme o synthase
**Cytochrome c oxidase, *cbb3* -type**	k255_56855	DJD_02940	*ccoO*	Cbb3-type cytochrome oxidase, cytochrome c subunit
	k255_56855	DJD_02941	*ccoN*	Cbb3-type cytochrome oxidase CcoN
	k255_56855	DJD_02942	*ccoP*	cytochrome c oxidase, cbb3-type, subunit III CcoP
	k255_56855	DJD_02945	*ccoP*	cytochrome c oxidase, cbb3-type, subunit III CcoP
**F-type ATPase**	k255_30799	DJD_02478	*atpF*	F-type ATP synthase, membrane subunit b
	k255_30799	DJD_02479	*atpF*	F-type ATP synthase, membrane subunit b
	k255_30799	DJD_02480	*atpH*	ATP synthase delta chain
	k255_30799	DJD_02481	*atpA*	ATP synthase alpha chain
	k255_30799	DJD_02482	*atpG*	ATP synthase gamma chain
	k255_30799	DJD_02483	*atpD*	ATP synthase beta chain
	k255_30799	DJD_02484	*atpC*	ATP synthase epsilon chain
	k255_72007	DJD_01086	*atpB*	ATP synthase F0 sector subunit a
	k255_72007	DJD_01087	*atpE*	ATP synthase F0 sector subunit c
**V-type ATPase**	k255_16252	DJD_01730	*atpE*	V-type ATP synthase subunit E
	k255_16252	DJD_01732	*atpA*	V-type ATP synthase subunit A
	k255_16252	DJD_01733	*atpB*	V-type ATP synthase subunit B

	k255_16252	DJD_01734	*atpD*	V-type ATP synthase subunit D
	k255_16252	DJD_01735	*atpI*	V-type ATP synthase subunit I
	k255_16252	DJD_01736	*atpK*	V-type ATP synthase subunit K
**Type I Bacterial secretion system**	k255_59323	DJD_02174	*tolC*	outer membrane protein
	k255_61013	DJD_02252	*tolC*	outer membrane protein
	k255_9420	DJD_03793	*tolC*	outer membrane protein
	k255_13259	DJD_02003	*hlyD*	membrane fusion protein, hemolysin D
	k255_61013	DJD_02250	*hlyB*	ATP-binding cassette, subfamily B, bacterial HlyB/CyaB
**Type II bacterial secretion system**	k255_10952	DJD_03573	*gspD*	Type II secretory pathway component GspD
	k255_10952	DJD_03581	*gspE*	Type II secretory pathway ATPase GspE
	k255_24565	DJD_00844	*gspC*	Type II secretory pathway, component PulC
	k255_24565	DJD_00845	*gspD*	Type II secretory pathway component GspD
	k255_33169	DJD_01303	*gspD*	Type II secretory pathway component GspD
	k255_33169	DJD_01307	*gspK*	Type II secretory pathway, general secretion pathway protein K
	k255_33169	DJD_01311	*gspF*	Type II secretory pathway, component PulF
	k255_33169	DJD_01312	*gspE*	Type II secretory pathway ATPase GspE
	k255_33169	DJD_01313	*gspG*	Type II secretory pathway, general secretion pathway protein G
	k255_33169	DJD_01445	*gspF*	Type II secretory pathway, component PulF
	k255_34859	DJD_02563	*secE*	Preprotein translocase subunit SecE
	k255_34859	DJD_02624	*secF*	Preprotein translocase subunit SecF
	k255_34859	DJD_02625	*secD*	Preprotein translocase subunit SecD
	k255_34859	DJD_02626	*yajC*	Preprotein translocase subunit YajC
	k255_39923	DJD_04634	*secG*	Preprotein translocase subunit SecG
	k255_41778	DJD_00758	*gspJ*	Type II secretory pathway, general secretion pathway protein J
	k255_41778	DJD_00759	*gspI*	Type II secretory pathway, general secretion pathway protein I
	k255_42243	DJD_02688	*secY*	Preprotein translocase subunit SecY
	k255_45298	DJD_02749	*gspK*	Type II secretory pathway, general secretion pathway protein K
	k255_45298	DJD_02750	*gspJ*	Type II secretory pathway, general secretion pathway protein J
	k255_9435	DJD_03895	*tatA*	Sec-independent protein translocase protein TatA
	k255_41604	DJD_04528	*tatA*	Sec-independent protein translocase protein TatA

	k255_45298	DJD_02765	*tatB*	Twin-arginine translocation protein TatB
	k255_45298	DJD_02766	*tatC*	Sec-independent protein secretion pathway component TatC
	k255_46175	DJD_01567	*ffh*	Signal recognition particle GTPase
	k255_47382	DJD_04653	*ftsY*	Signal recognition particle GTPase
	k255_51998	DJD_01464	*gspK*	Type II secretory pathway, general secretion pathway protein K
	k255_57676	DJD_01847	*gspG*	Type II secretory pathway, general secretion pathway protein G
	k255_58849	DJD_04805	*gspG*	Type II secretory pathway, general secretion pathway protein G
	k255_73408	DJD_00882	*gspM*	Type II secretory pathway, general secretion pathway protein M
	k255_13259	DJD_02005	*yidC*	Membrane protein insertase YidC
	k255_9435	DJD_03905	*gspH*	Type II secretory pathway, general secretion pathway protein H
	k255_9435	DJD_03906	*gspF*	Type II secretory pathway, component PulF
	k255_9435	DJD_03909	*gspE*	Type II secretory pathway ATPase GspE
	k255_9435	DJD_03908	*gspE*	Type II secretory pathway ATPase GspE
	k255_14910	DJD_03061	*secA*	Preprotein translocase subunit SecA (ATPase, RNA helicase)
**MAGs and iron transport**	k255_73582	DJD_02412	*mad26*	magnetosome protein Mad26
	k255_73582	DJD_02413	*mad25*	magnetosome protein Mad25
	k255_73582	DJD_02414	*mad24*	magnetosome protein Mad24
	k255_73582	DJD_02415	*mad23*	magnetosome protein Mad23
	k255_73582	DJD_02416	*mamB*	Magnetosome protein MamB
	k255_73582	DJD_02417	*mamO*	magnetosome protein MamO-Cter
	k255_73582	DJD_02418	*man6*	magnetosome protein Man6
	k255_73582	DJD_02419	*man5*	magnetosome protein Man5
	k255_73582	DJD_02420	*man4*	magnetosome protein Man4
	k255_73582	DJD_02421	*mamQ*	magnetosome protein MamQ-1
	k255_73582	DJD_02422	*mamE*	magnetosome protein MamE
	k255_73582	DJD_02423	*mamI*	magnetosome protein MamI
	k255_73582	DJD_02424		unknown gene
	k255_73582	DJD_02425	*mamA*	magnetosome protein MamA
	k255_73582	DJD_02426	*mad2*	magnetosome protein Mad2
	k255_73582	DJD_02427	*mamB*	magnetosome protein MamB

	k255_73582	DJD_02428	*mamQ*	magnetosome protein MamQ-2
	k255_73582	DJD_02429	*mad31*	magnetosome protein Mad31
	k255_73582	DJD_02430	*mamM*	magnetosome protein MamM
	k255_73582	DJD_02431	*mamP*	magnetosome protein MamP
	k255_73582	DJD_02432	*man3*	magnetosome protein Man3
	k255_73582	DJD_02433	*mad10*	magnetosome protein Mad10
	k255_73582	DJD_02434	*man2*	magnetosome protein Man2
	k255_73582	DJD_02435	*man1*	magnetosome protein Man1
	k255_12352	DJD_03158	*mad30*	magnetosome protein Mad30
	k255_12352	DJD_03159	*mad17*	magnetosome protein Mad17
	k255_12352	DJD_03160		unknown gene
	k255_12352	DJD_03161	*mad28*	magnetosome protein Mad28
	k255_72157	DJD_01620	*feoB*	Fe^{2+} transport system protein FeoB
	k255_72157	DJD_01621	*feoA*	Fe^{2+} transport system protein FeoA
	k255_72157	DJD_01623	*feoB*	Fe^{2+} transport system protein FeoB
	k255_72157	DJD_01624	*feoA*	Fe^{2+} transport system protein FeoA
	k255_12370	DJD_03175	*tonB*	periplasmic protein TonB
	k255_12370	DJD_03176	*exbD*	biopolymer transport protein ExbD
	k255_12370	DJD_03177	*exbB*	biopolymer transport protein ExbB
	k255_12370	DJD_03178	*exbB*	biopolymer transport protein ExbB
	k255_12370	DJD_03183	—	TonB-dependent copper receptor
**Circadian clock protein**	k255_58849	DJD_04861		Phosphoacceptor receiver (REC) domain
	k255_58849	DJD_04860	*kaiB*	Circadian clock protein KaiB3
	k255_58849	DJD_04859	*kaiC*	Circadian clock protein kinase KaiC3
	k255_58849	DJD_04858		Phosphoacceptor receiver (REC) domain
	k255_58849	DJD_04857	*kaiB*	Circadian clock protein KaiB2
	k255_58849	DJD_04856	*kaiC*	Circadian clock protein kinase KaiC2
	k255_58849	DJD_04855		Signal transduction histidine kinase domain
	k255_58849	DJD_04854		Response regulator
	k255_58849	DJD_04853		Phosphoacceptor receiver (REC) domain and histidine kinase domain

k255_58849	DJD_04852		3'3'-cGAMP-specific phosphodiesterase
k255_58849	DJD_04851		DNA binding response regulator and histidine kinase domain
k255_58849	DJD_04850		DNA binding response regulator and histidine kinase domain
k255_58849	DJD_04849		Substrate-binding domain-containing protein
k255_58849	DJD_04848		Histidine kinase domain
k255_58849	DJD_04847		Signal histidine kinase domain
k255_58849	DJD_04846		Pyruvate, phosphate dikinase
k255_58849	DJD_04845		Hypothetical protein
k255_58849	DJD_04844		Response regulator receiver domain and EAL domain
k255_58849	DJD_04843		REC domain, PAS domain and EAL domain
k255_58849	DJD_04842		REC domian
k255_58849	DJD_04841	*kaiB*	Circadian clock protein KaiB1
k255_58849	DJD_04840	*kaiC*	Circadian clock protein kinase KaiC1
k255_58849	DJD_04839	*kaiB*	Circadian clock protein KaiB4
k255_58849	DJD_04838		Signal transduction histidine kinase domain
k255_58849	DJD_04383	*kaiC*	Circadian clock protein kinase KaiC4
k255_15989	DJD_00611	*kaiB*	Circadian clock protein KaiB5
k255_15989	DJD_00610	*kaiC*	Circadian clock protein kinase KaiC5
k255_15989	DJD_00609		GAF domain

Table S4 List of the sequence information involved in the KaiC phylogenetic analysis

Organisms	Genbank assembly accession	KaiC protein accession
Caballeronia temeraria strain LMG 29319	GCA_001544795.2	SAK55138.1, SAK56770.1, SAK60653.1, SAK74601.1, SAK74672.1
Xanthomonas hortorum ASM228551v1	GCA_002285515.1	ASW44587.1
Paraburkholderia phenoliruptrix BR3459a	GCA_000300095.1	WP_014972880.1
Variovorax paradoxus B4	GCA_000463015.1	AGU48855.1
Duganella phyllosphaerae strain T54	GCA_001758785.1	WP_070247717.1
Saccharolobus solfataricus strain SULM	GCA_003852155.1	WP_009988518.1, WP_269454403.1
Synechocystis sp. PCC 6803	GCA_000009725.1	BAA18762.1, BAA18607.1, BAA17922.1
Pyrobaculum aerophilum str. IM2	GCA_000007225.1	WP_011009172.1
Pyrococcus horikoshii OT3	GCA_000011105.1	WP_048053076.1
Aeropyrum pernix strain K1	GCA_000011125.1	WP_010866413.1
Thermosynechococcus vestitus BP-1	GCA_000011345.1	WP_011056334.1
Prochlorococcus marinus subsp. pastoris str. CCMP1986	GCA_000011465.1	WP 011132976.1
Rippkaea orientalis PCC 8801	GCA_000021805.1	WP_015785209.1, WP_012597132.1
Methanocaldococcus jannaschii DSM 2661	GCA_000091665.1	WP_010870876.1
Chroococcidiopsis thermalis PCC 7203	GCA_000317125.1	WP_015155513.1
Cylindrospermum stagnale PCC 7417	GCA_000317535.1	WP_015208186.1, WP_015208712.1
Synechococcus elongatus PCC 6301	GCA_000010065.1	WP 011242648.1
Nostoc sp. PCC 7120	GCA_000009705.1	BAB74585.1
Candidatus Magnetominusculus xianensis HCH-1	GCA_001541255.1	KWT91952.1, KWT78287.1
Candidatus Magnetobacterium cryptolimnobacter XYR	GCA_018606785.1	GCA_018606785.1, GCA_018606785.1
Candidatus Magnetobacterium casensis MYR-1	GCA_000714715.1	WP_040335196.1, WP_162176222.1
Candidatus Magnetoovum chiemensis CS-04	GCA_000960725.1	KJR42959.1, KJR42335.1
***Candidatus* Magnetocorallium paracelsis XS-1**	**JAREWP000000000**	**DJD_00610, DJD_04383, DJD_04840, DJD_04856, DJD_04859**
Candidatus Magnetoglobus multicellularis str. Araruama	GCA_000516475.1	ETR68795.1, ETR71667.1, ETR71669.1
Candidatus Magnetomorum sp. HK-1	GCA_001292585.1	KPA10379.1, KPA16384.1, KPA16386.1, KPA17301.1, KPA19595.1, KPA19597.1
Candidatus Magnetananas sp. SF-1	not published	ID:52102585, ID:52106710, ID:52106711
