# Supplementary figures and images for: Insight into the metabolic potential and ecological function of a novel Magnetotactic Nitrospirota in coral reef habitat
Source: Front Microbiol. 2023 May 17;14:1182330. doi: 10.3389/fmicb.2023.1182330 (PMC10278575; doi:10.3389/fmicb.2023.1182330)

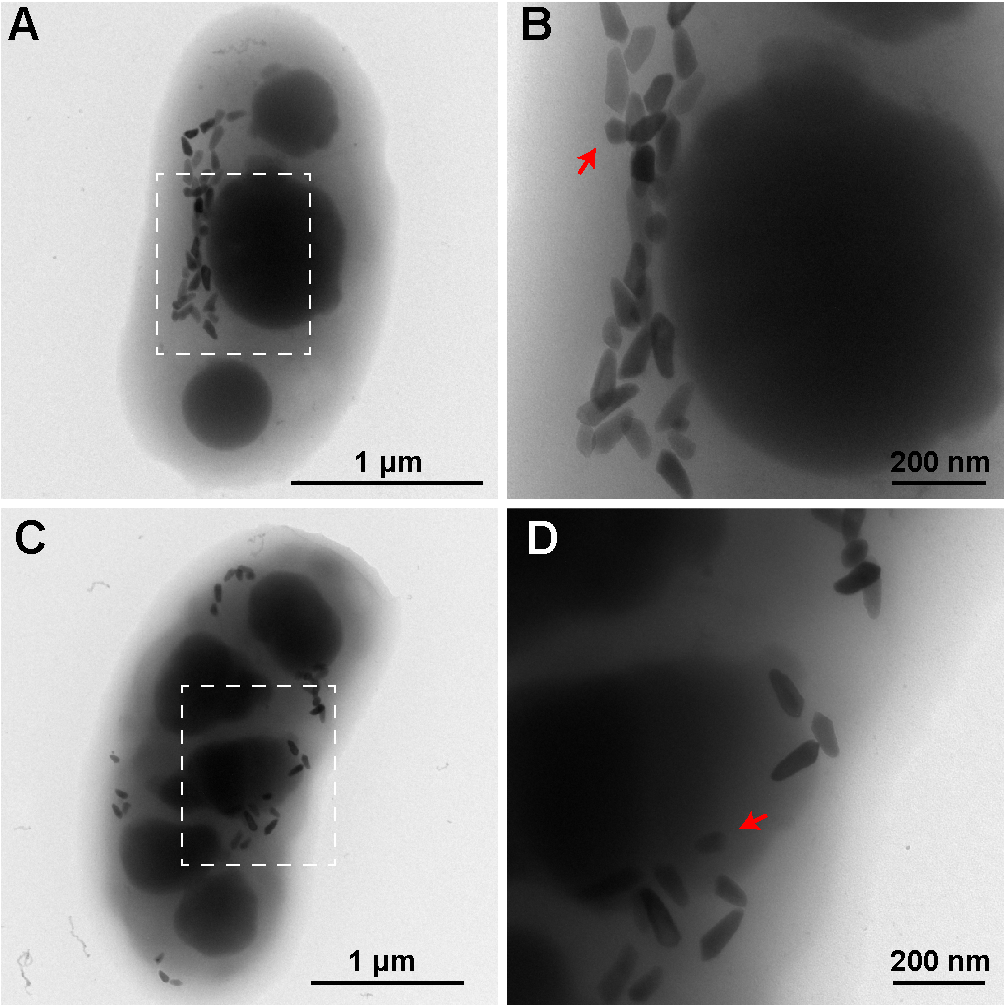

Supplement: Supplementary Figure S1 — Transmission electron micrographs of XS-1. (A) TEM images showing bullet-shaped magnetosomes loosely arranged in bundles roughly lying along the long cell axis. (C) TEM images showing bullet-shaped magnetosomes scattered in the XS-1 cell. (B) and (D) are the magnified images of the area marked by dash line rectangle in (A) and (C), respectively. Red arrows indicate the immature magnetosomes. [file Image_1.tif]

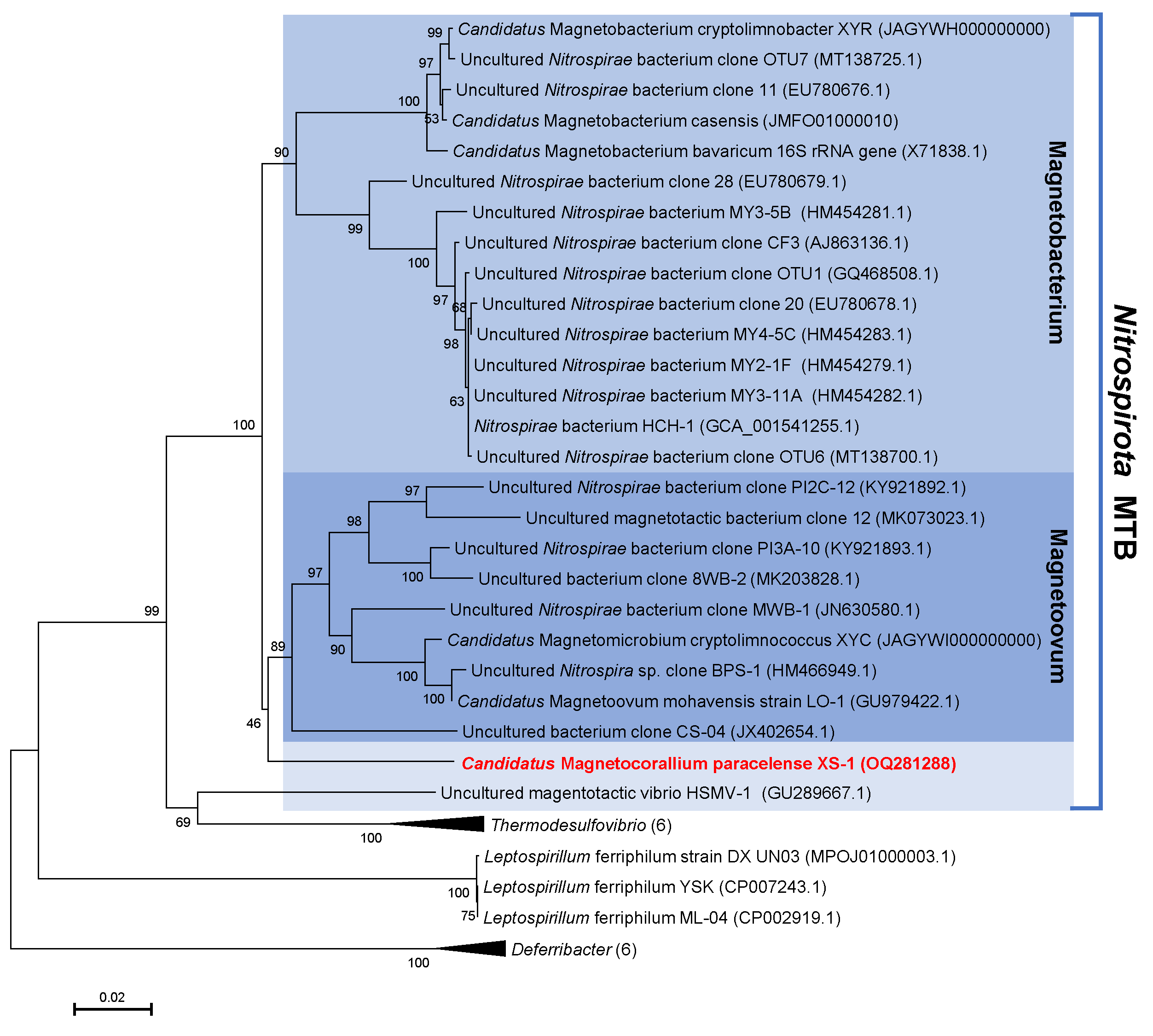

Supplement: Supplementary Figure S2 — Neighbor-joining tree based on 16S rRNA gene sequences. The multiple sequence alignment consisting of 16S rRNA genes contained Nitrospirota MTB and non-MTB, rooted with representative members of the Deferribacterota phylum. The bootstrap support values (out of 1000 replicates) are indicated as percentages. The sequence in this study is highlighted in red. [file Image_2.tif]
